# Hybrid whale optimization algorithm for enhanced routing of limited capacity vehicles in supply chain management

**DOI:** 10.1038/s41598-024-51359-2

**Published:** 2024-01-08

**Authors:** Vu Hong Son Pham, Van Nam Nguyen, Nghiep Trinh Nguyen Dang

**Affiliations:** Department of Construction Engineering and Management, Ho Chi Minh City University of Technology (HCMUT), Vietnam National University (VNU-HCM), Ho Chi Minh City, Vietnam

**Keywords:** Mathematics and computing, Applied mathematics, Computer science, Engineering, Civil engineering, Experimental evolution

## Abstract

The present study focuses on the problem of vehicle routing with limited capacity, with the objective of minimizing the transportation distance required to serve h clients with predetermined locations and needs. The aim is to create k trips that cover the shortest possible distance. To achieve this goal, a hybrid whale optimization algorithm (hGWOA) is proposed, which combines the whale optimization algorithm (WOA) with the grey wolf optimizer (GWO). The proposed hybrid model is comprised of two main steps. First step, the GWO’s hunting mechanism is integrated transitioning to the utilization phase of WOA, and a newly devised state is introduced that is linked to GWO. In the second step, a novel technique is incorporated into the exploration mission phase to enhance the resolve after per iteration. The algorithm’s performance is assessed and compared with other modern algorithms, including the GWO, WOA, ant lion optimizer (ALO), and dragonfly algorithm (DA) using 23 benchmark test functions and CEC2017 benchmark test function. The results indicate that the hybrid hGWOA method outperforms other algorithms in terms of delivery distance optimization for scenarios involving scale and complexity. These findings are corroborated through case studies related to cement delivery and a real-world scenario in Viet Nam.

## Introduction

The vehicle routing problem (VRP) with limited capacity serves as a complex extension of the classic traveling salesman problem (TSP). In this context, the objective is to outline *k* routes, optimizing for minimal cost or distance, to cater to ℎ clients, each with their predetermined locations and demands. It’s crucial that each vehicle starts and concludes its journey at a specified point, all while adhering to particular constraints. Numerous methodologies have been proposed to tackle the VRP challenge. These include linear programming, the ant lion optimizer (ALO), particle swarm optimization (PSO), modified hybrid particle swarm optimization (MHPSO), double population genetic algorithm (DPGA), whale optimization algorithm (WOA), grey wolf optimizer (GWO), genetic algorithm (GA), and the dragonfly algorithm (DA).

In the realm of transportation and logistics, the VRP stands as a paradigmatic NP-hard challenge. Despite being the subject of extensive academic investigation, characterizing the VRP remains elusive due to its intricate array of constraints and stipulations. These include factors like Chronological Span, Length, Collection and Drop-off, and Capability, as outlined by Laporte^1^. As a result, research endeavors addressing the VRP are tasked with focusing on pivotal parameters such as length^2^, cost^3^, and the intertwined factors of temporal duration and carbon emissions^4^. Liu et al.^5^ differentiated the VRP from the TSP by highlighting the former's provision for multiple routes. Each of these routes is constrained by a specific vehicle capacity and must traverse all nodes. Given the daunting complexity inherent to the VRP, research has chiefly gravitated towards heuristic and meta-heuristic strategies as the primary methodologies to derive workable solutions.

The VRP has ascended as a key subject in academic research, chiefly due to its pivotal role in transportation and logistics. Given the necessity to ensure punctual deliveries of large volumes of goods, the task often exceeds the capabilities of individual vehicles. Taking into account each vehicle's inherent capacity and load restrictions, devising astute delivery routes becomes essential to meet daily consumer demands. Researchers in this arena endeavor to calibrate the objective function, targeting an optimal solution that simultaneously minimizes costs, geographical span, time constraints, and carbon emissions. Such optimization efforts encompass a range of approaches, from tackling the VRP in contexts where goods are dispatched from a single depot^6,7^ to more intricate setups originating from multiple depots^8,9^.

The significance of optimization is evident across a myriad of fields, leading to a marked increase in the focus on metaheuristic techniques. One of the salient features of metaheuristics is their adaptability. From a broader perspective, metaheuristics can be delineated based on the degree of randomness they introduce during each optimization iteration. They can also be characterized based on their foundational inspirations, many of which are derived from swarm intelligence. Examples include the whale optimization algorithm (WOA)^10^, the grey wolf optimizer (GWO)^11^, and the African wild dog optimization algorithm (AWDO)^12^. These metaheuristic techniques find applications in diverse domains, such as the time–cost trade-off in construction projects^13,14^, dispatching of ready-mix concrete trucks^15^, optimization of construction site layouts^16^, VRP^17^, reduction of construction material costs^18^, logistics cost optimization^19^, and the design optimization of water distribution systems^20^.

The WOA, a metaheuristic optimization technique, was introduced by Mirjalili and Lewis^10^ in 2016. Deriving its inspiration from the intricate hunting behaviors of humpback whales, this method employs a set of candidate solutions, each representing a potential optimum. The WOA unfolds through a three-pronged schema of search strategies: exploration, exploitation, and convergence. In the exploration phase, the algorithm adopts a stochastic approach, identifying promising regions within the vast search space. As it shifts to the exploitation stage, it mirrors the humpback's bubble-net hunting tactics to close in on these pinpointed regions. Finally, in the convergence phase, the WOA concentrates on the fine-tuning of the best solution, progressively narrowing the search scope. Despite inherent limitations, such as sensitivity to parameter variations and a tendency towards premature convergence, the WOA is lauded for its versatility, user-friendly nature, and notable efficiency in diverse sectors, including engineering, electrical systems, and finance.

The WOA has garnered significant attention due to its wide applicability across diverse domains. Correspondingly, there has been a surge in research initiatives aimed at refining its optimization capabilities. Chakraborty, Saha^21^ unveiled a modified WOA (mWOAPR) to enhance the diagnosis of COVID-19 severity using chest X-ray images. Notably, their findings outperformed both the foundational and other advanced metaheuristic algorithms, especially in benchmarking and segmenting COVID-19 X-ray images. A subsequent study proposed an elite-based WOA variant (EBWOA)^22^, addressing certain limitations of the conventional WOA. This iteration demonstrated its efficacy across benchmark functions, IEEE CEC 2019 functions, design issues, and tangible cloud scheduling dilemmas. An additional WOA modification, optimized for high-dimensional problems, was introduced^23^, addressing challenges like inadequate exploration, compromised accuracy, and premature convergence. Building upon prior research, Chakraborty, Saha^24^ enriched the WOA (designated WOAmM) by incorporating a revised mutualism phase from the Symbiotic Organisms Search (SOS) algorithm. This enhancement specifically targeted premature convergence pitfalls. In a distinct development, a novel WOA iteration (m-SDWOA) was put forth^25^ amalgamating features from both the SOS and Differential Evolution (DE). This fusion harmoniously married exploration and exploitation, culminating in improved accuracy, diversity, and mitigation of early convergence. In another collaborative effort, Chakraborty, Sharma^26^ rolled out an optimized WOA version (ImWOA) with aspirations to magnify diversity, exploration, and solution precision. Their evaluations rendered promising results across a spectrum of optimization tasks, including image segmentation, particularly when benchmarked against rudimentary algorithms and newer WOA iterations. Lastly, a fusion of success-history-based adaptive differential evolution (SHADE) with a customized WOA was presented^27^, culminating in the SHADE-WOA hybrid. This avant-garde optimization technique manifested exemplary results, both in standard benchmarks and practical engineering design tasks, as corroborated by comprehensive statistical examinations.

In 2014, Mirjalili et al.^11^ pioneered the GWO, a metaheuristic approach inspired by the behavioral dynamics of grey wolves. When compared to prevailing metaheuristic techniques like PSO, DE, GSA, and FEP^28^, GWO stands out, particularly during its exploitation phase. The algorithm demonstrates an innate ability to deftly navigate the solution space, outperforming in avoiding local optima in a significant majority of the 29 functions examined^11^. Nevertheless, the GWO, despite its astute update mechanism, is not devoid of challenges. Researchers have identified difficulties in balancing exploration and exploitation^29^ and pointed out its limited success in tackling issues related to non-linear equation systems and unconstrained optimization^29^. This underscores the pressing need to further refine and enhance GWO to overcome these intrinsic shortcomings. While attempts to diversify the population through random initialization of the grey wolves’ population have been made, such a strategy is not without pitfalls—a concern later addressed^30^.

The main issues with WOA, as identified in^28–31^, have motivated the authors of this paper to propose a hybridized approach using GWO. This hybrid method starts by initializing the initial population according to WOA, GWO to create population diversity and sort preliminary results. Next, the study proposes to use the leadership hierarchy inherent in GWO to apply WOA’s bubble attack strategy. In the mining phase, the proposed algorithm selected the top three alpha, beta, and delta wolves from the entire search agent, and the other search agents modified their positions according to the agent’s position. find the best of other search agents to improve the performance of the WOA algorithm through the p-factor. The performance goals of hGWOA are demonstrated through unilateral and multimodal benchmark functions. This solved the local optimization problems, incomplete solution improvement after each iteration, and low performance in the exploitation phase of WOA.

The large-scale capacity vehicle routing problem (CVRP) stands at the crux of effective transportation and logistics management. Though myriad solutions have been proposed to address this problem, the performance of many such methodologies in confronting extensive CVRPs leaves much to be desired. Bridging this gap, we present the hGWOA, a state-of-the-art hybrid optimizer. By seamlessly fusing the strengths of the GWO and WOA, the hGWOA promises to deliver potent solutions specifically tailored for medium to large-scale CVRPs, and beyond this, for a diverse range of optimization challenges inherent in real-world transportation systems. The deployment of this innovative model not only accentuates optimization efficacy but also empowers decision-makers, bestowing upon them the capability to derive astute, well-informed, and strategic solutions that address the complex nuances of transportation logistics.

The remains of this study are structured as follow: section “Literature review” provides a comprehensive review of the existing literature on the vehicle routing problem. In section “Model development”, we present the specifics of our proposed hybrid grey wolf optimizer algorithm. Section “Computational experiments” evaluates the algorithm’s performance and effectiveness in comparison to existing models. Lastly, section “Conclusion” concludes our findings, highlighting the study’s contributions and suggesting potential areas for future research in the field.

## Literature review

The VRP has been the subject of rigorous investigation for over six decades, with a plethora of strategies and objectives proposed^32–35^. One prevalent approach for addressing the VRP factors is in both distance and customer demands. This strategy employs the “3-opt” framework in tandem with mixed-integer linear programming for uniformly sized vehicles, and binary linear programming when dealing with fleets of varied sizes^2^. Additionally, research endeavors in this arena have delved into optimizing processes like the loading and unloading of goods^2^, refining travel and service intervals^36^, curtailing operational costs such as vehicular wear, fuel consumption, and refrigeration expenses^3^, and emphasizing the reduction of carbon footprints^4,5^.

Capacity limitations are frequently observed in various research studies, acting as a fundamental constraint in vehicle routing problems. The CVRP has been the subject of numerous methodologies developed to address its complexities. These methodologies are broadly classified into two categories: exact methods and heuristic methods, each possessing its own unique attributes and advantages. The ant colony algorithm (ACO) was first introduced by Dorigo et al.^37^ as a simulation-based optimization technique that mirrors the food-seeking behavior of real-world ants. This algorithm has been widely employed to address the travelling salesman problem (TSP) and other intricate combinatorial challenges. The fundamental premise of the ACO is that the paths traversed by ants represent potential solutions to the optimization dilemma. As time progresses, there is a systematic increase in the concentration of pheromones on the more optimal paths. Consequently, a higher number of ants are inclined to select shorter routes, paving the way to pinpointing the optimal solution. To enhance the efficiency of the ACO, various adaptations have been suggested by researchers. Notably, Dorigo et al.^37^ proposed the ant colony system (ACS) as a refined version of the original algorithm. Moreover, Yu et al.^38^ presented an augmented ACO equipped with an intensified local search capability. Furthering the innovations in this field, Chen and Shi^39^ put forward a hybrid methodology that melds local search techniques with the foundational principles of the ant colony algorithm, specifically targeting the multi-compartment vehicle routing challenge.

The CVRP has captivated the attention of researchers aiming to augment the efficacy of transportation systems. A plethora of algorithms addressing this conundrum have been proposed, including contributions by Pham and Nguyen^17^, Azad^40^. Korayem et al. ^41^ introduced an inventive approach that amalgamates K-means clustering with grey wolf optimization, aiming for adept group formation and routing. On a similar note, Ng et al.^42^ unveiled the multiple-colonies artificial bee colony methodology, which employs a re-routing paradigm to optimize CVRP solutions. Another notable contribution is by Wei et al.^43,44^ who infused two-dimensional packing constraints into the Simulated Annealing framework for CVRP problem-solving. This adaptation not only modifies the neighborhood structure but also augments the solution’s quality. They further expanded on this by developing a method that accentuated CVRP optimization through the integration of two-dimensional packing constraints. Delving into more intricate challenges, Tao and Wang^45^ tackled the three-dimensional loading CVRP (3L-CVRP) by embedding three-dimensional packing and loading capacity constraints within the tabu search algorithm. In a parallel stride, Zhang et al.^46^ devised a random local search technique focusing on the same constraints. Both research endeavors furnish competent solutions for the 3L-CVRP, underscoring distinct search strategies tailored to specific constraints. Akpinar^47^ championed a hybrid approach, harnessing the strengths of both large-scale neighborhood search and ant colony algorithms to refine the optimization process. Furthermore, Sze et al.^48^ presented a two-phase hybrid approach with an adjustable locality mechanism, embedding a large neighborhood search to diversify the solution pool. In another noteworthy contribution, Akhand et al.^49^ integrated adaptive scanning and velocity speculation into the particle swarm optimization (PSO) technique, enhancing path optimization. They further honed the PSO method, tailoring it for the optimization of garbage collection routes. Collectively, these methodologies illuminate pathways for refining transport systems, providing robust solutions that bolster transportation operations’ efficiency.

Reed et al.^50^ employed ACS to devise routing strategies for vehicles in cyberspace. They further broadened its application by integrating multi-chambered vehicles designed for waste sorting. Remarkably, their methodology led to a significant cost reduction of 15% in a management science project undertaken at E. I. Du Pont, Inc^34^. In another innovative approach, Narasimha et al.^51^ presented a VRP formulation centered on minimizing the journey time of the vehicle traversing the longest route. This perspective is especially pertinent in situations demanding rapid emergency responses. Furthermore, a subset of scholars has broadened the scope of VRP models to incorporate diverse parameters. These include customer satisfaction, environmental emissions, and cost optimization^7,8^ and^52^;

Amidst rising apprehensions regarding global warming, the mitigation of carbon emissions has taken center stage in the discourse on the VRP. In response to these environmental challenges, many nations have instated taxes predicated on the carbon emissions produced by transport vehicles. This has underscored the imperative of cultivating efficient solutions to address these emission concerns. Consequently, there has been a marked surge in research endeavors over recent years, focusing on optimizing carbon emissions within the context of the VRP^5,53^. Given the intricate nature and expansive scale of the VRP, the quest for optimal resolutions often relies on heuristic and meta-heuristic methodologies. Such strategies are pivotal in sculpting efficient and environmentally sustainable transportation frameworks.

Beyond the scope of the traditional VRP, the dynamic vehicle routing problem (DVRP) has emerged as a significant area of interest. In the DVRP, new orders surface while goods are in transit, necessitating real-time route modifications^54^. To address this dynamic challenge, researchers have turned to strategies such as the PSO method and adaptive neighborhood search. Moreover, in a bid to minimize carbon emissions, the MDGVR problem has been introduced. This problem centers around eco-friendly vehicles that commence their routes from various depots but conclude at a singular, primary warehouse^9^. A proposed resolution for this particular challenge hinges on the deployment of a two-stage ACS methodology.

This research presents a new methodology, denoted as hGWOA, crafted to tackle the distance optimization challenges inherent to CVRP, aiming to reduce associated logistics expenses. To ascertain the efficacy of hGWOA, it was juxtaposed with several established algorithms, namely GWO, independent WOA, DA, and ALO. This comparative analysis utilized both classical benchmark test functions and CEC2017 test functions. The results underscore that hGWOA's performance is notably superior to its counterparts. Following this, the hGWOA algorithm was employed on two emblematic CVRP scenarios, further elucidated in section “Computational experiments”.

## Model development

### CVRP description and mathematical model

In the domain of operations research and logistics, the CVRP problem's significance is widely acknowledged^55^. This problem centers on crafting an optimal plan for transporting goods from a central warehouse to a set group of clients using a vehicle fleet, with the subsequent return of the fleet to the base. Shan and Wang^56^ have clearly defined this challenge, emphasizing two key constraints: firstly, the strict carrying capacity of each cargo vehicle, ensuring the total goods volume or weight on any given route does not exceed the vehicle's limits; and secondly, the requirement for each client to be visited only once, ensuring efficient and timely deliveries. The overarching goal of the CVRP is to minimize the entire journey distance of the fleet during its operations^17^.

Consider:

$$ \begin{gathered}   D = total~\;distance\;~travelled~\;by\;~all\;~units \hfill \\   x_{{ijt}}  = \left\{ {\begin{array}{*{20}c}    {1,~\;vehicle\;~t~\;depart\;~from\;~i~\;to~\;j}  \\    {0,~\;otherwise}  \\   \end{array} } \right.;y_{{it}}  = \left\{ {\begin{array}{*{20}c}    {1,~\;customer\;~i~\;is~\;served\;~by\;~unit~\;t}  \\    {0,~\;otherwise}  \\   \end{array} } \right. \hfill \\  \end{gathered}  $$

Objective function:

1 $$ Min D = \mathop \sum \limits_{i = 0}^{k} \mathop \sum \limits_{j = 0}^{k} \mathop \sum \limits_{t = 1}^{h} c_{ij} x_{ijt} $$

2 $$ \mathop \sum \limits_{i = 0}^{k} x_{ijt} = y_{jt} ;j = 1,2, \ldots ,k;t = 1,2, \ldots ,h $$

3 $$ \mathop \sum \limits_{i = 0}^{k} x_{ijt} = y_{it} ;j = 1,2, \ldots ,k;t = 1,2, \ldots ,h $$

4 $$ \mathop \sum \limits_{i = 0}^{k} g_{i} y_{it} \le q_{t} y_{it} ;t = 1,2, \ldots ,h $$

5 $$ \mathop \sum \limits_{t = 1}^{h} y_{it} = \left\{ {\begin{array}{*{20}c} {1; i = 1,2,3, \ldots ,k} \\ {h;i = 0} \\ \end{array} } \right\} $$

where *c*_*ij*_ represents the cost from customer *i* to customer *j*. The symbol *g*_*i*_ stands for the demand of the *i*th client, with *i* taking values from 1 through *k*, where *k* is the total number of clients. The letter *h* represents the total number of units. Lastly, *q*_*t*_ indicates the capacity of the *t*th unit, with *t* ranging from 1 to *h*.

Equation (1) defines the objective function for the VRP. Within this equation, *x*_*ijt*_ is a binary variable indicating the route's selection status. It is assigned a value of 1 if the route is chosen and 0 otherwise. The VRP's primary goal is to minimize the cumulative distance traveled, epitomized by the sum of the distances covered by each unit. Equations (2) and (3) are constraints ensuring that there’s a unique path linking each unit to every client. Specifically, Eq. (2) mandates that each client is visited only once, whereas Eq. (3) stipulates that each unit must visit a minimum of one client. The unit capacity constraint is introduced in Eq. (4), restricting the volume of goods transported along a particular route. The sum of goods delivered to every client along a route must stay within the unit’s designated capacity. Lastly, **Eq. **(5) dictates that a singular unit exclusively services each client. In contrast, the warehouse receives attention from *h* units, where *h* denotes the specific number of units assigned to the warehouse.

### Hybrid whale optimization algorithm model for CVRP

#### Whale Optimization Algorithm_WOA

**Figure 1:**
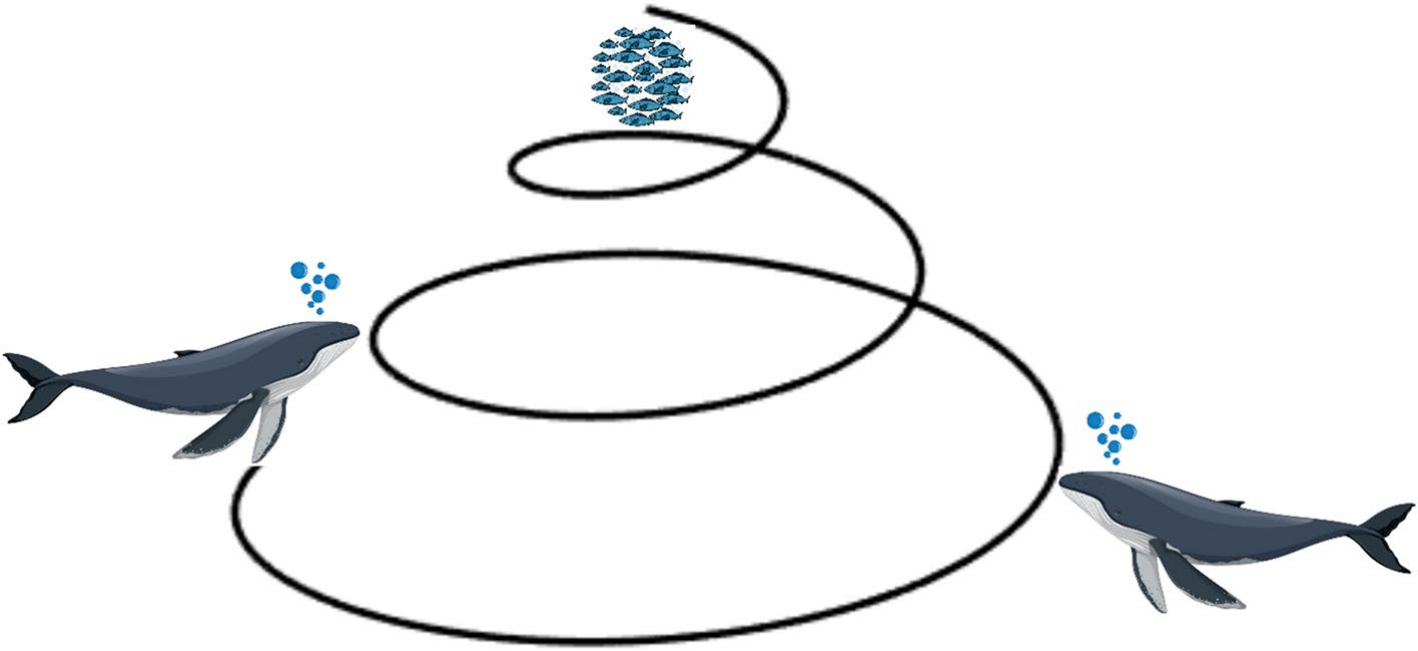
Bubble-net feeding strategy of humpback whales.

In 2016, Mirjalili and Lewis^10^ unveiled the WOA, a pioneering metaheuristic optimization technique. Inspired by the intricate hunting behaviors of humpback whales, the WOA facilitates proficient exploration and exploitation of the search space to pinpoint optimal solutions. As illustrated in Fig. 1, the WOA operationalizes through three distinct phases: encircling the prey, navigating the spiral bubble trap, and the subsequent prey hunt.

##### Encircling prey

Humpback whales have a unique ability to detect and encircle their prey. However, given that the exact position of the optimal solution within the search space remains unknown a priori, the WOA algorithm predicates the notion that the current best candidate solution either signifies the target prey or is in proximity to the optimal solution. Upon the identification of the best-performing search agent, the other agents endeavor to recalibrate their positions in alignment with this top-scoring agent. This behavior is encapsulated mathematically in Eqs. (6) and (7):

6 $$ \vec{D} = \left| {\vec{C} \times \vec{X}^{*} \left( t \right) - \vec{X}\left( t \right)} \right| $$

7 $$ \vec{X}\left( {t + 1} \right) = \vec{X}^{*} \left( t \right) - \vec{A} \times \vec{D} $$

In the Eqs. (8) and (9), the term *t* stands for the current iteration. $$\vec{A}$$ and $$\vec{C}$$ are known as coefficient vectors. $${X}^{*}$$ indicates the position vector of the most optimal solution found until the present iteration, while $$X$$ signifies the position vector of the current search agent. The || represent the concept of absolute value. It's important to highlight that $$ X^{*}$$ needs updating every iteration if a better solution emerges.

The calculation for the vectors $\vec{A}$ and $\vec{C}$ is as follows:

8 $$ \vec{A} = 2\vec{a} \times \vec{r} - \vec{a} $$

9 $$ \vec{C} = 2 \times \vec{r} $$

where *a* undergoes a decremental variation, starting from an initial value of 2 and culminating at a value of 0 as the iterations ensue. This decrement is manifest in both the exploration and exploitation phases. In addition, the variable *r* represents a vector whose elements are randomly generated, with values ranging between 0 and 1.

**Figure 2:**
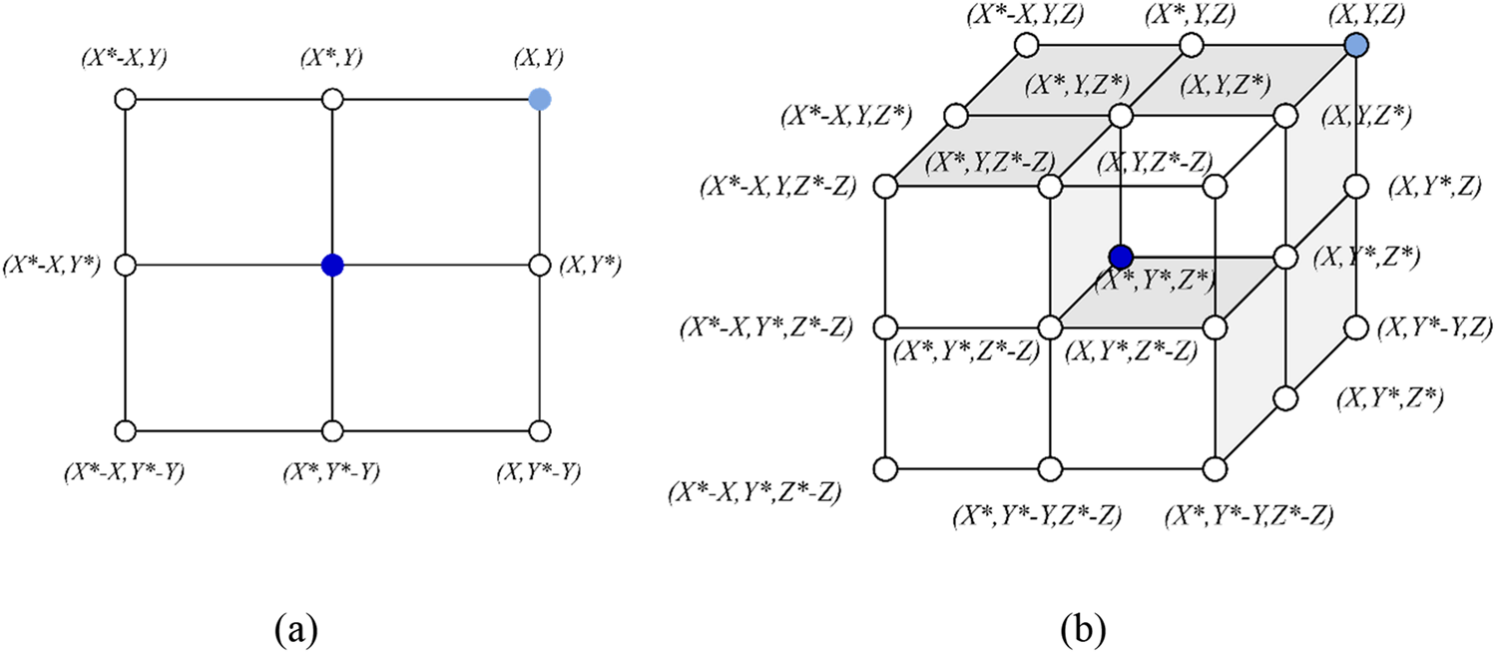
2D and 3D position vectors and their possible subsequent placements (X^*^ is the top-performing solution obtained so far).

Figure 2a offers a graphical illustration of the application of Eq. (7) to a two-dimensional problem. It elucidates the method by which a search agent's position is updated in relation to the most recent solution's position. Through modifications to the vectors $\vec{A}$ and $\vec{C}$, the search agent can traverse various regions proximate to the highest-performing solution. Figure 2b extrapolates this notion to a three-dimensional context, highlighting the potential update trajectories of a search agent. Importantly, the random vector ($\overrightarrow{r}$) empowers the search agent to probe any location within the search domain, as delineated by the pivotal points in Fig. 2. As a result, Eq. (7) aids in refining a search agent’s position near the apex-performing solution, simulating the dynamics of encircling prey.

##### Bubble-net attacking method (exploitation phase)

**Figure 3:**
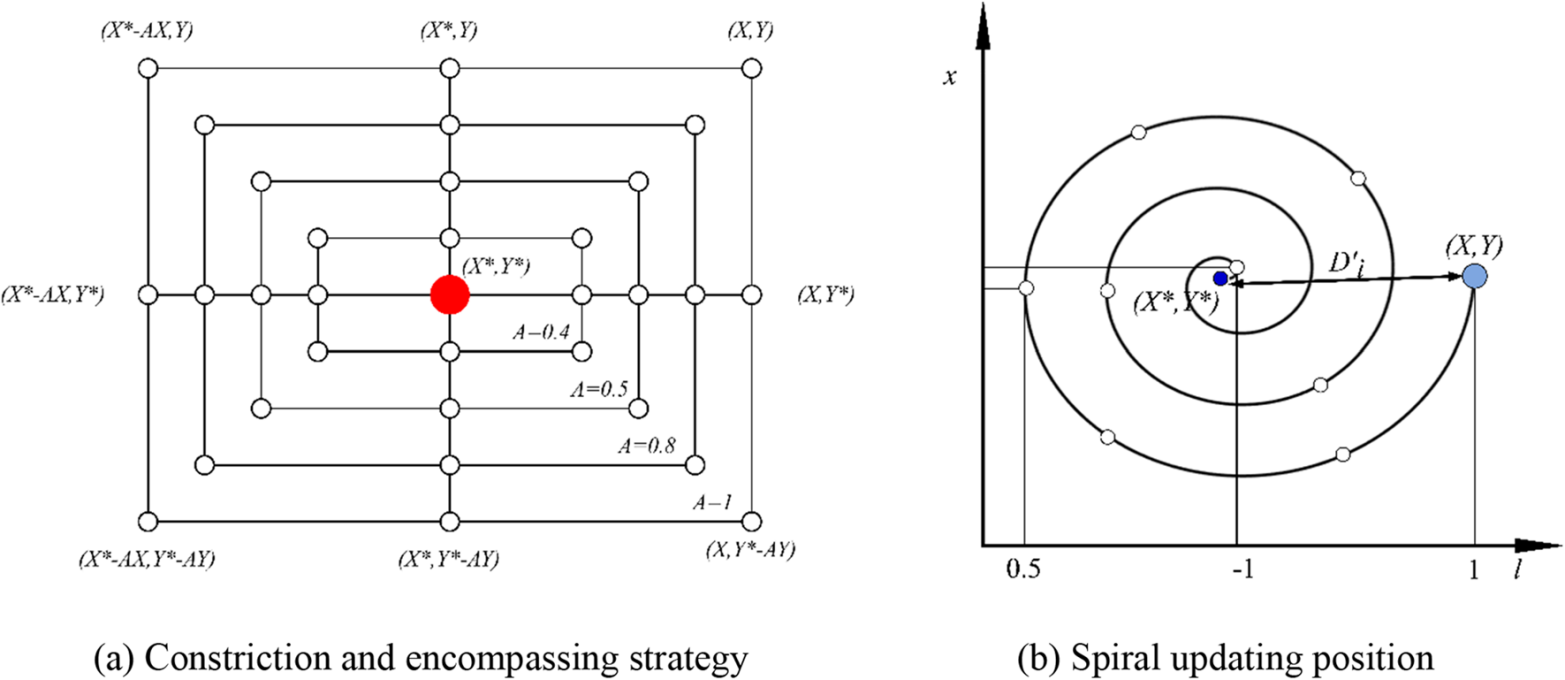
Bubble-net search mechanism implemented in WOA (X ^*^is the top-performing solution).

To formulate a mathematical representation of the bubble-net foraging tactics observed in humpback whales, two distinct methodologies have been proposed:

- *Constriction and Encompassing Strategy:* This approach endeavors to emulate the behavior through modifications to the parameter and vectors in Eq. (8). Specifically, the magnitude of ‘a’ is diminished, which consequently reduces the variation amplitude of $\overrightarrow{A}$. Here, $\overrightarrow{A}$ is an unpredictable value confined to the interval *[− a, a]*. As the iterations progress, the value of *a* is systematically reduced from 2 to 0. By assigning random values to $\overrightarrow{A}$ within the range of *[− 1, 1]*, it becomes feasible to position a search agent anywhere between its originating position and the location of the best-performing agent. Figure 3a graphically illustrates the potential positions that can be achieved within a 2D plane, spanning from $(X, Y)$ to $({X}^{*}, {Y}^{*})$, contingent on the constraint $0\le A\le 1$.
- *Spiral Updating Position Approach*: As illustrated in Fig. 3b, this methodology commences by computing the Euclidean distance between the whale's position $\left( {X, Y} \right)$ and its prey's position $\left( {X^{*} , Y^{*} } \right)$. The subsequent step involves devising a spiral equation, designed to mimic the helical trajectory often exhibited by humpback whales as they converge on their target. The derived equation is articulated as:
  10 $$ \vec{X}\left( {t + 1} \right) = \vec{D} \times e^{bl} \times \cos \left( {2\pi l} \right) + \vec{X}^{*} \left( t \right) $$
  11 $$ \vec{D} = \left| {\vec{X}^{*} \left( t \right) - \vec{X}\left( t \right)} \right| $$

In Eqs. (10) and (11), the vectors $\vec{D}$ and the variable *l* denote the distance between the *i*th whale and the prey. These serve dual purposes: first, as indicators of the spatial proximity between the two entities, and second, as metrics to gauge the quality of the optimal solution acquired up to that point. The constant *b* emerges as a pivotal element, endowing the logarithmic spiral with its unique characteristics. Furthermore, the variable *l* is derived from a uniform distribution over the interval *[− 1, 1]*, infusing the equation with a stochastic component.

The collective behavior of humpback whales, characterized by their tendency to encircle prey in a narrowing loop while also adopting a spiral trajectory, is emulated in the model. Within this framework, a balanced probability of 50% is designated to either the contraction-encircling mechanism or the spiral model. This probabilistic approach dictates how the whales' positions are updated throughout the optimization procedure. The mathematical articulation of this model is presented as follows:

12 $$ \vec{X}\left( {t + 1} \right) = \left\{ {\begin{array}{*{20}l} {\vec{X}^{*} \left( t \right) - \vec{A} \times \vec{D}} \hfill & {if \;p < 0.5} \hfill \\ {\vec{D} \times e^{bl} \times \cos \left( {2\pi l} \right) + \vec{X}^{*} \left( t \right)} \hfill & {if \;p \ge 0.5 } \hfill \\ \end{array} } \right. $$

A similar method, centered on the manipulation of vector ${\vec{\text{A}}}$, finds application in the pursuit of prey during the exploration phase. In this context, humpback whales engage in stochastic search behaviors influenced by the relative positions of their peers. Consequently, vector $\overrightarrow{{\text{A}}}$ is endowed with random values exceeding 1 or descending below − 1, serving to compel a search agent to undertake substantial displacements from a reference whale. Diverging from the exploitation phase, where a search agent's position is updated based on the most successful agent discovered thus far, the exploration phase employs a different strategy. Here, the updating of a search agent's position hinges on the random selection of another search agent, rather than relying on the best-found agent. This mechanism, when coupled with $\left| A \right| > 1$, underscores the significance of exploration, thereby empowering the WOA to conduct an extensive global search. The mathematical formulation is presented as follows:

13 $$ \vec{D} = \left| {\vec{C} \times \vec{X}_{rand} - \vec{X}} \right| $$

14 $$ \vec{X}\left( {t + 1} \right) = \vec{X}_{rand} - \vec{A} \times \vec{D} $$

where $\vec{X}_{rand}$ denotes a stochastic position vector, which is selected from the existing population of whales.

### Grey Wolf Optimizer (GWO)

**Figure 4:**
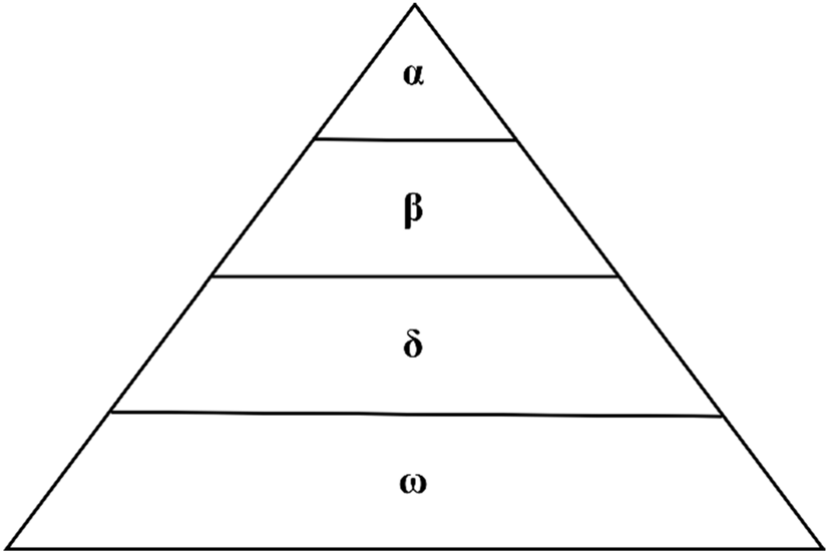
Grey wolf population organization chart.

The GWO algorithm was introduced by Mirjalili et al.^11^ in 2014, drawing inspiration from the hunting and hierarchical leadership behavior of wild wolves. The algorithm comprises four levels, denoted as alpha, beta, delta, and omega. In this hierarchy, the first three wolves represent the best variants within the population, while omega (*ω*) symbolizes the variation within the population, as illustrated in Fig. 4. Additionally, the algorithm models the two distinct stages of the wolf population: the siege stage and the hunt for prey stage.

The siege phase is displayed as follows:

15 $$ \vec{d} = \left| {\vec{c} \times \vec{c}_{p}^{t} - \vec{x}^{t} } \right| $$

16 $$ \vec{x}^{{\left( {t + 1} \right)}} = \vec{x}^{t} - \vec{a} \times \vec{d} $$

where $\vec{x}^{t}$ is the wolf's position in iteration *t*, $\vec{d}{ }$ is the prey’s position vector, $\vec{a}$ and $\vec{c}$ represents coefficient vectors, which are computed as follows:

17 $$ \vec{a} = 2l \times r_{1} $$

18 $$ \vec{c} = 2 \times r_{2} $$

During the hunting phase, Mirjalili models the hunting behavior by assuming that alpha, beta, and delta have knowledge of the potential position of the prey based on their experience. This is expressed mathematically as follows:

19 $$ \vec{d}_{\alpha } = \left| {\vec{c}_{1} \times \vec{x}_{\alpha } - \vec{x}} \right|;\vec{d}_{\beta } = \left| {\vec{c}_{2} \times \vec{x}_{\beta } - \vec{x}} \right|;\vec{d}_{\delta } = \left| {\vec{c}_{3} \times \vec{x}_{\delta } - \vec{x}} \right| $$

20 $$ \vec{x}_{1} = \vec{x}_{\alpha } - \vec{a}_{1} \times \vec{d}_{\alpha } ; \vec{x}_{2} = \vec{x}_{\beta } - \vec{a}_{2} \times \vec{d}_{\beta } ; \vec{x}_{3} = \vec{x}_{\delta } - \vec{a}_{3} \times \vec{d}_{\delta } $$

21 $$ \vec{x}^{{\left( {t + 1} \right)}} = \frac{{\vec{x}_{1} + \vec{x}_{2} + \vec{x}_{3} }}{3} $$

During the search and attack phase, a vector $\vec{a}$ is randomly generated within the range of [-2a, 2a]. If $\left| {\vec{a}} \right| < 1$, the wolves will attack a randomly selected prey, referred to as the mining stage. However, if $\left| {\vec{a}} \right| > 1$, the wolves may abandon their current target and search for better prey^57^. Another parameter that influences the decoy search is the variable c, which takes a value within the range of^2^. A vector $\vec{c}$ is randomly and abruptly updated to prevent local optimization. If $c > 1$, the solution will converge towards the prey, whereas if $c < 1$, the solution will move away from the prey in search of new targets.

### Hybrid whale optimization algorithm model for CVRP

This section introduces a proposed methodology that combines the WOA and the GWO to enhance the efficiency of the WOA during its exploitation phase. This aims to attain superior solutions, drawing upon the insights discussed in the preceding sections regarding both WOA and GWO.

Despite the standard version of the WOA exhibiting a notable proficiency in identifying optimal solutions, its capability to consistently refine these solutions in subsequent iterations might be limited. To address this limitation and bolster the WOA’s performance, an amalgamation with the GWO was proposed, leading to the genesis of a novel algorithm termed hGWOA. This innovative hybridization introduces two pivotal modifications to the conventional WOA. Foremost, a conditional constraint is embedded within WOA's exploitation phase, aiming to augment its hunting efficacy.As illustrated by Eq. (21), the parameters $\vec{x}_{1}$, $\vec{x}_{2}$, and $\vec{x}_{3}$ are pivotal to the exploitation performance of the GWO. To circumvent the challenges of local optima, particularly when each ‘a’ is either less than 1 or greater than − 1, a novel condition has been incorporated into hGWOA’s standard exploitation phase. Furthermore, modifications have been made to Eqs. (19), (20), and (21) to facilitate their use within this newly introduced condition, focusing expressly on the parameters $\vec{x}_{1}$, $\vec{x}_{2}$, and $\vec{x}_{3}$. In addition, a supplementary criterion has been introduced during the exploration phase of hGWOA to guide the current solution more effectively towards the most propitious outcome, while concurrently forestalling the whale from advancing to a position inferior to its preceding location.

hGWOA initiates by establishing a population comprising search agents, encompassing both whales and wolves. This population is subsequently subjected to a procedure designed to rectify agent positions that surpass the defined boundaries of the search space. Following this positional adjustment, the fitness function is meticulously computed for each agent. In instances where an agent's fitness falls below the alpha_score (best_score), the alpha_score is updated to align with the agent's computed fitness. Consequently, pivotal algorithmic variables, including *a, A, C, L* and *p* are subject to updates, and a random *p* number is stochastically generated.When the generated random number *p* falls below the threshold of 0.5, it triggers an evaluation of an additional conditional statement, which inquires whether *|A|* does not equal 1. If this condition is met, a new position for the agent is computed utilizing Eq. (6). Subsequent to this calculation, if the fitness of the newly derived position surpasses that of the current position, the algorithm updates the agent's position accordingly. However, if the condition *|A|*≥ *1* holds true, then the new position is determined utilizing Eq. (7). Analogous to the prior condition, the algorithm scrutinizes the fitness of the new position relative to the old, and if superiority is established, the agent's position undergoes a corresponding update. In an alternative scenario, if the randomly generated variable *p* is greater than or equal to 0.5, and all the variables *a1*, a*2* and *a3* fall within the range of − 1 to 1, then the algorithm proceeds to update the current solution's position utilizing Eq. (21).

Following these steps, the algorithm checks if any newly computed positions exceed the defined search space limits. If they do, corrective actions are taken to bring them within bounds. This process results in the calculation of updated fitness values for the agents, ultimately leading to the identification and reporting of the algorithm’s optimal fitness achievement.

**Figure 5:**
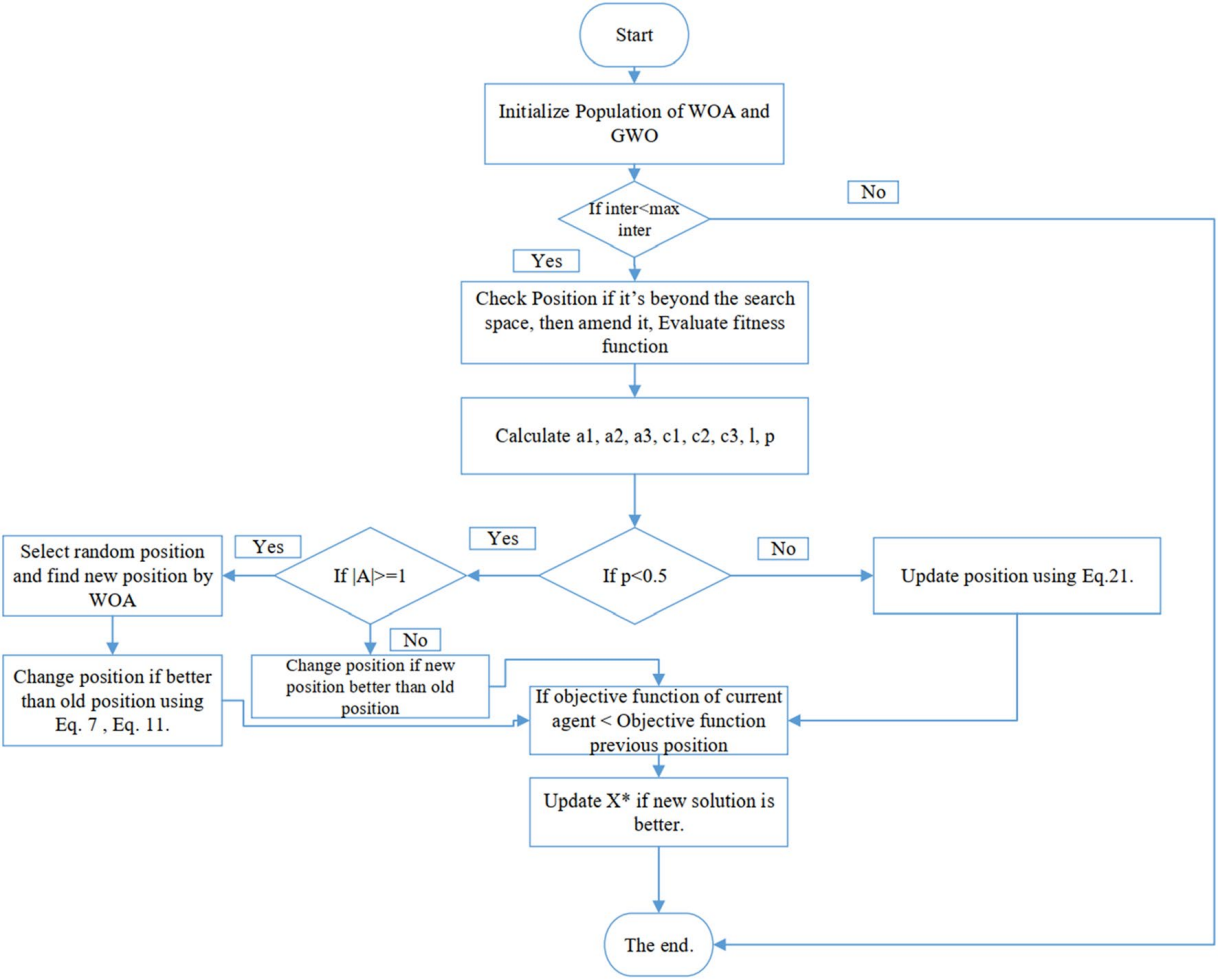
Flowchart of the proposed hGWOA method.

The fundamental distinction between WOA and hGWOA is observed in the incorporation of Eqs. (19), (20), and (21) during the exploitation phase of WOA. This is further complemented by an innovative strategy introduced in the exploration stage to enhance the solution quality. The integration of these equations, coupled with this new strategy, amplifies the foraging efficiency of WOA. As a result, the optimal solution undergoes refinement in each iteration, bolstering the algorithm's resilience against local optima. Additionally, the introduction of this specific condition during the exploration phase augments the algorithm's search capability, reinforcing the robustness of existing solutions. Table 1 summarizes the parameters used, demonstrating an appropriate blend for the hGWOA, WOA, and GWO algorithms. Concurrently, Table 2 and Fig. 5 present the pseudo-code and flowchart for the hGWOA approach, respectively.

**Table 1:** Parameter settings of the hGWO, GWO and WOA.

Algorithm	Parameter	Value
hGWOA	Population size	60
	Number of Iterations	500
	Time taken by each function to complete	30
GWO	Population size	60
	Number of Iterations	500
	Time taken by each function to complete	30
WOA	Population size	60
	Number of Iterations	500
	Time taken by each function to complete	30

**Table 2:** Pseudo-code of the proposed hGWOA method.

**Step 1:** Generate a population of hGWOA population by *Xi (i* = *1, 2, 3, 4 …, n)* **Step 2:** Evaluate the fitness of each solution **Step 3:** Set X* as the solution with the highest fitness **Step 4:** Repeat the following steps while the number of iterations is less than the maximum: **Step 5:** For each solution , update the variables *a, A, C, l*, and *p* **Step 6:** If p is less than 0.5: a. If the absolute value of *A* is less than 1, update the position of the current whale using Eq. (6) b. If the current fitness is better than the previous fitness, set the position to the new position **Step 7:** Else if the absolute value of *A* is greater than or equal to 1, select a random whale and update the position using Eq. (13) a. If the current fitness is better than the previous fitness, set the position to the new position **Step 8:** Else if *p i*s greater than or equal to 0.5 and all variables *a1, a2*, and *a3* are between -1 and 1, update the location of the current whale using Eq. (21) **Step 9:** Check if any whale has gone beyond the search space and adjust its position if necessary **Step 10:** Evaluate the fitness of each whale **Step 11:** If there is a whale with better fitness than X*, update X* **Step 12:** Increase the iteration counter t by 1 **Step 13:** Return X*

The hGWOA algorithm showcases significant advancements in integrating both global and local search strategies within the search space. This hybrid approach generates a succession of stochastic solutions during its initial phase, optimizing the quest for the ideal solution. Additionally, the hGWOA methodology utilizes an iterative framework, enabling the effective pinpointing and harnessing of unexplored regions within the search domain. Consequently, this leads to the revelation of novel and promising solutions.

## Computational experiments

### Convergence behaviours on classical benchmark function

A detailed evaluation of the hGWOA’s optimization prowess was executed, using classical benchmark test functions that are widely acknowledged in the field. Comparative analyses pitted hGWOA against four prominent optimization methodologies: GWO, WOA, DA, and ALO. The benchmark test functions deployed in this study were categorized based on their distinctive traits into three groups: uni-modal, multi-modal, and fixed-dimensional composite functions with multiple local optima. Comprehensive depictions of these functions can be found in Tables 3, 4 and 5.

**Table 3:** Uni-modal test functions.

Function	Dim	Range	fmin
$f1\left( x \right) = \mathop \sum \limits_{i = 1}^{n} x_{i}^{2}$	10	[− 100, 100]	0
$f2\left( x \right) = \mathop \sum \limits_{i = 1}^{n} \left| {x_{i} } \right| + \mathop \prod \limits_{i = 1}^{n} \left| {x_{i} } \right|$	10	[− 10, 10]	0
$f3\left( x \right) = \mathop \sum \limits_{i = 1}^{n} \left( {\mathop \sum \limits_{j - 1}^{i} x_{j} } \right)^{2}$	10	[− 100, 100]	0
$f4\left( x \right) = max\left\{ {\left| {x_{i} } \right|, 1 \le i \le n} \right\}$	10	[− 100, 100]	0
$f5\left( x \right) = \mathop \sum \limits_{i = 1}^{n - 1} \left[ {100\left( {x_{i + 1} - x_{i}^{2} } \right)^{2} + \left( {x_{i} - 1} \right)^{2} } \right]$	10	[-30,30]	0
$f6\left( x \right) = \mathop \sum \limits_{i = 1}^{n} \left( {\left| {x_{i} + 0.5} \right|} \right)^{2}$	10	[− 100, 100]	0
$f7\left( x \right) = \mathop \sum \limits_{i = 1}^{n} ix_{i}^{4} + random\left[ {0,1} \right)$	10	[− 1.28, 1.28]	0

**Table 4:** Multi-modal test functions.

Function	Dim	Range	fmin
$f8\left( x \right) = \mathop \sum \limits_{i = 1}^{n} - x_{i} {\text{sin}}\left( {\sqrt {\left| {x_{i} } \right|} } \right)$	10	[− 500, 500]	− 2820.8
$f9\left( x \right) = \mathop \sum \limits_{i = 1}^{n} \left[ {x_{i}^{2} - 10\cos \left( {2\pi x_{i} } \right) + 10} \right]$	10	[− 5.12, 5.12]	0
$f10\left( x \right) = - 20\exp \left( { - 0.2\sqrt {\frac{1}{n}\mathop \sum \limits_{i = 1}^{n} x_{i}^{2} } } \right) - exp\left( {\frac{1}{n}\mathop \sum \limits_{i = 1}^{n} \cos \left( {2\pi x_{i} } \right)} \right) + 20 + e$	10	[− 32, 32]	0
$f11\left( x \right) = \frac{1}{4000}\mathop \sum \limits_{i = 1}^{n} x_{i}^{2} - \mathop \prod \limits_{i = 1}^{n} \cos \left( {\frac{{x_{i} }}{\sqrt i }} \right) + 1$	10	[− 600, 600]	0
$f12\left( x \right) = \frac{\pi }{n}\left\{ {10\sin^{2} \left( {\pi y_{1} } \right) + \mathop \sum \limits_{i = 1}^{n} \left( {y_{i} - 1} \right)^{2} \left[ {1 + 10\sin^{2} \left( {\pi y_{i + 1} } \right)} \right] + \left( {y_{n} - 1} \right)^{2} + \mathop \sum \limits_{i = 1}^{n} u\left( {x_{i} ,10,100,4} \right)} \right\}$ $y_{i} = 1 + \frac{{x_{i} + 1}}{4}$ $$u\left( {x_{i} ,a,k,m} \right) = \left\{ {\begin{array}{*{20}c} {k\left( {x_{i} - a} \right)^{m} x_{i} > a} \\ {0 - a < x_{i} < a} \\ {k\left( { - x_{i} - a} \right)^{m} x_{i} < - a} \\ \end{array} } \right.$$	10	[− 50, 50]	0
$f13\left( x \right) = 0.1\left\{ {\sin^{2} \left( {3\pi x_{1} } \right) + \mathop \sum \limits_{i = 1}^{n} \left( {x_{i} - 1} \right)^{2} \left[ {1 + \sin^{2} \left( {3\pi x_{i} + 1} \right)} \right] + \left( {x_{n} - 1} \right)^{2} [1 + \sin^{2} \left( {2\pi x_{n} } \right)]} \right\} + \mathop \sum \limits_{i = 1}^{n} u\left( {x_{i} ,5,100,4} \right)$	10	[− 50, 50]	0

**Table 5:** Fixed functions with multiple local optima.

Function	Dim	Range	fmin
$f14\left( x \right) = \left( {\frac{1}{500} + \mathop \sum \limits_{j = 1}^{25} \frac{1}{{j + \mathop \sum \nolimits_{i = 1}^{2} \left( {x_{i} - a_{ij} } \right)^{6} }}} \right)^{ - 1}$	2	[− 65, 65]	1
$f15\left( x \right) = \mathop \sum \limits_{i = 1}^{11} \left[ {a_{i} - \frac{{x_{1} \left( {b_{i}^{2} + b_{i} x_{2} } \right)}}{{b_{i}^{2} + b_{i} x_{3} + x_{4} }}} \right]^{2}$	4	[− 5, 5]	0.0003
$f16\left( x \right) = 4x_{1}^{2} - 2.1x_{1}^{4} + \frac{1}{3}x_{1}^{6} + x_{1} x_{2} - 4x_{2}^{2} + 4x_{2}^{4}$	2	[− 5, 5]	− 1.0316
$f17\left( x \right) = \left( {x_{2} - \frac{5.1}{{4\pi^{2} }}x_{1}^{2} + \frac{5}{\pi }x_{1} - 6} \right)^{2} + 10\left( {1 - \frac{1}{8\pi }} \right)cosx_{1} + 10$	2	[− 5, 5]	0.398
$f18\left( x \right) = \left[ {1 + \left( {x_{1} + x_{2} + 1} \right)^{2} \left( {19 - 14x_{1} + 3x_{1}^{2} - 14x_{2} + 6x_{1} x_{2} + 3x_{2}^{2} } \right)} \right] \times \left[ {30 + \left( {2x_{1} - 3x_{2} } \right)^{2} \times \left( {18 - 32x_{1} + 12x_{1}^{2} + 48x_{2} - 36x_{1} x_{2} + 27x_{2}^{2} } \right)} \right]$	2	[− 2, 2]	3
$f19\left( x \right) = - \mathop \sum \limits_{i = 1}^{4} c_{i} exp\left( { - \mathop \sum \limits_{j = 1}^{3} a_{ij} \left( {x_{j} - p_{ij} } \right)^{2} } \right)$	3	[0, 1]	− 3.86
$f20\left( x \right) = - \mathop \sum \limits_{i = 1}^{4} c_{i} exp\left( { - \mathop \sum \limits_{j = 1}^{6} a_{ij} \left( {x_{j} - p_{ij} } \right)^{2} } \right)$	6	[0, 1]	− 3.32
$f21\left( x \right) = - \mathop \sum \limits_{i = 1}^{5} \left[ {\left( {X - a_{i} } \right)\left( {X - a_{i} } \right)^{T} + c_{i} } \right]^{ - 1}$	4	[0, 10]	− 10.1532
$f22\left( x \right) = - \mathop \sum \limits_{i = 1}^{7} \left[ {\left( {X - a_{i} } \right)\left( {X - a_{i} } \right)^{T} + c_{i} } \right]^{ - 1}$	4	[0, 10]	− 10.4028
$f23\left( x \right) = - \mathop \sum \limits_{i = 1}^{10} \left[ {\left( {X - a_{i} } \right)\left( {X - a_{i} } \right)^{T} + c_{i} } \right]^{ - 1}$	4	[0, 10]	− 10.5363

For a rigorous and impartial comparative analysis, each algorithm was run 30 times for every benchmark function. Following this, statistical evaluations were conducted to determine both the central tendency and variability of data from these 30 runs. The research framework utilized 60 search agents, each limited to a maximum of 500 iterations. Tables 6, 7 and 8 present the statistical results, encompassing mean values (*ave*) and standard deviations (*std*), of the hGWOA approach, comparing its performance to other notable algorithms, including DA, ALO, GWO, and WOA.

**Table 6:** Results of different algorithms on uni-modal functions.

F	hGWOA		GWO		WOA		DA		ALO	
	Ave	Std	Ave	Std	Ave	Std	Ave	Std	Ave	Std
F1	7.81E−61	1.37E−60	1.64E−57	5.11E−57	1.42E−57	7.57E−57	1.07E+01	2.01E+01	3.39E−08	4.33E−08
F2	9.87E−43	1.75E−42	1.05E−40	2.43E−40	1.45E−40	5.48E−40	1.72E+00	1.26E+00	6.27E−05	3.55E−05
F3	1.37E−28	1.41E−28	4.85E+04	1.46E+04	4.98E+04	1.53E+04	1.65E+02	1.60E+02	4.59E+00	1.55E+01
F4	3.33E+01	2.95E+01	4.83E+01	3.09E+01	5.86E+01	2.02E+01	3.00E+00	1.70E+00	7.09E−02	3.29E−01
F5	6.09E+00	2.94E−01	2.82E+01	4.07E−01	2.82E+01	3.51E−01	9.49E+02	1.24E+03	1.73E+02	4.05E+02
F6	2.17E−06	5.25E−07	5.80E−01	2.57E−01	7.04E−01	3.27E−01	1.01E+01	2.28E+01	4.93E−08	7.41E−08
F7	2.33E−04	8.19E−05	4.40E−03	5.92E−03	5.55E−03	5.64E−03	3.60E−02	3.41E−02	3.63E−02	2.30E−02

**Table 7:** Results of different algorithms on multi-model functions.

F	hGWOA		GWO		WOA		DA		ALO	
	Ave	Std	Ave	Std	Ave	Std	Ave	Std	Ave	Std
F8	− 1.04E+04	1.89E+03	− 1.04E+04	1.67E+03	− 1.00E+04	1.78E+03	− 2.82E+03	3.59E+02	− 2.26E+03	4.83E+02
F9	0.00E+00	0.00E+00	0.00E+00	0.00E+00	0.00E+00	0.00E+00	2.49E+01	1.19E+01	2.04E+01	1.03E+01
F10	3.61E−15	1.50E−15	5.63E−15	2.95E−15	4.68E−15	3.03E−15	3.22E+00	1.18E+00	5.97E−01	8.45E−01
F11	3.14E−03	4.83E−03	7.82E−03	4.21E−02	1.40E−02	5.31E−02	5.65E−01	3.76E−01	2.18E−01	1.03E−01
F12	3.78E−02	1.78E−02	3.49E−02	1.76E−02	1.80E−01	8.18E−01	1.77E+00	1.56E+00	3.41E+00	3.31E+00
F13	1.34E−02	4.27E−02	6.84E−01	3.05E−01	6.89E−01	2.50E−01	2.12E+00	2.81E+00	3.56E−03	7.34E−03

**Table 8:** Results of different algorithms on fixed functions.

F	hGWOA		GWO		WOA		DA		ALO	
	ave	std	ave	std	ave	std	ave	std	ave	std
F14	9.98E−01	1.08E−10	3.36E+00	3.09E+00	2.96E+00	3.21E+00	1.26E+00	6.74E−01	3.78E+00	3.09E+00
F15	4.02E−04	3.64E−05	7.86E−04	4.80E−04	7.94E−04	5.41E−04	4.06E−03	6.39E−03	4.00E−03	6.51E−03
F16	-1.03E+00	1.03E−08	− 1.03E+00	5.64E−09	− 1.03E+00	2.46E−09	− 1.03E+00	1.10E−06	− 1.03E+00	1.85E−13
F17	3.98E−01	7.95E−08	3.98E−01	3.39E−05	3.98E−01	2.46E−05	3.98E−01	1.80E−08	3.98E−01	1.14E−13
F18	3.00E+00	6.42E−07	3.00E+00	1.04E−04	3.00E+00	1.10E−03	3.00E+00	1.68E−05	3.00E+00	5.83E−13
F19	− 3.86E+00	4.86E−06	− 3.85E+00	2.59E−02	− 3.85E+00	1.03E−02	− 3.86E+00	9.95E−04	− 3.86E+00	1.27E−12
F20	− 3.32E+00	4.04E−06	− 3.26E+00	9.58E−02	− 3.22E+00	1.60E−01	− 3.22E+00	8.84E−02	− 3.28E+00	5.68E−02
F21	− 8.28E+00	2.43E+00	− 8.18E+00	2.80E+00	− 7.53E+00	2.88E+00	− 6.94E+00	2.43E+00	− 5.97E+00	3.31E+00
F22	− 8.43E+00	2.55E+00	− 7.32E+00	3.12E+00	− 7.26E+00	3.22E+00	− 7.79E+00	2.80E+00	− 5.52E+00	3.10E+00
F23	− 7.99E+00	2.88E+00	− 6.89E+00	3.25E+00	− 6.13E+00	3.21E+00	− 7.06E+00	3.16E+00	− 5.74E+00	3.28E+00

It is imperative to highlight that uni-modal functions are characterized by a singular global extremum, making them an ideal benchmark for evaluating an algorithm’s capability in exploiting the search space. Upon examination of the results presented in Table 6, it is discernible that hGWOA surpasses other nature-inspired algorithms, namely ALO, DA, WOA, and GWO, in the domain of uni-modal mathematical functions. This superiority is evidenced by its consistent performance across all seven instances for GWO, WOA, and DA, and in six of the seven cases for ALO.

In contrast to uni-modal functions, multi-modal functions are distinguished by the presence of a singular optimal global point accompanied by multiple local optima. These characteristics make multi-modal functions particularly apt benchmarks for assessing the search space exploration competence of hGWOA. A close examination of the outcomes from the multi-modal test functions, as presented in Table 7, underscores hGWOA's superior performance relative to WOA, GWO, ALO and DA. Notably, hGWOA's efficacy surpasses that of DA across all six instances, outperforms WOA in four of the six, eclipses ALO in five of the six, and bests GWO in three of the six scenarios. Such outcomes attest to hGWOA's skill in adeptly navigating around local optima and its thorough probing of the search space. This exceptional performance accentuates the algorithm’s potential significance in academic research, particularly in the domain of exhaustive search space exploration.

Composite benchmark test functions represent an integration of various monomodal and multi-modal functions, subjected to transformations and perturbations, including rotation, translation, and bias. These composite benchmark evaluation functions share a consistent actual search domain replete with numerous local optima. This makes them particularly beneficial for assessing the balance between exploration and exploitation within the search space. Table 8 showcases the results of evaluating the efficacy of the hGWOA algorithm in addressing synthesized benchmark evaluation challenges (F14–F23). Based on the empirical findings, it can be inferred that the hGWOA algorithm surpasses other population-based optimization techniques in efficiency, underlining its prowess in striking an equilibrium between search space exploration and exploitation. This competency is further illuminated by the algorithm's aptitude to consistently demonstrate superior mean values, illustrating its balanced approach to the tradE−off between discovering and harnessing the search space.

**Figure 6:**
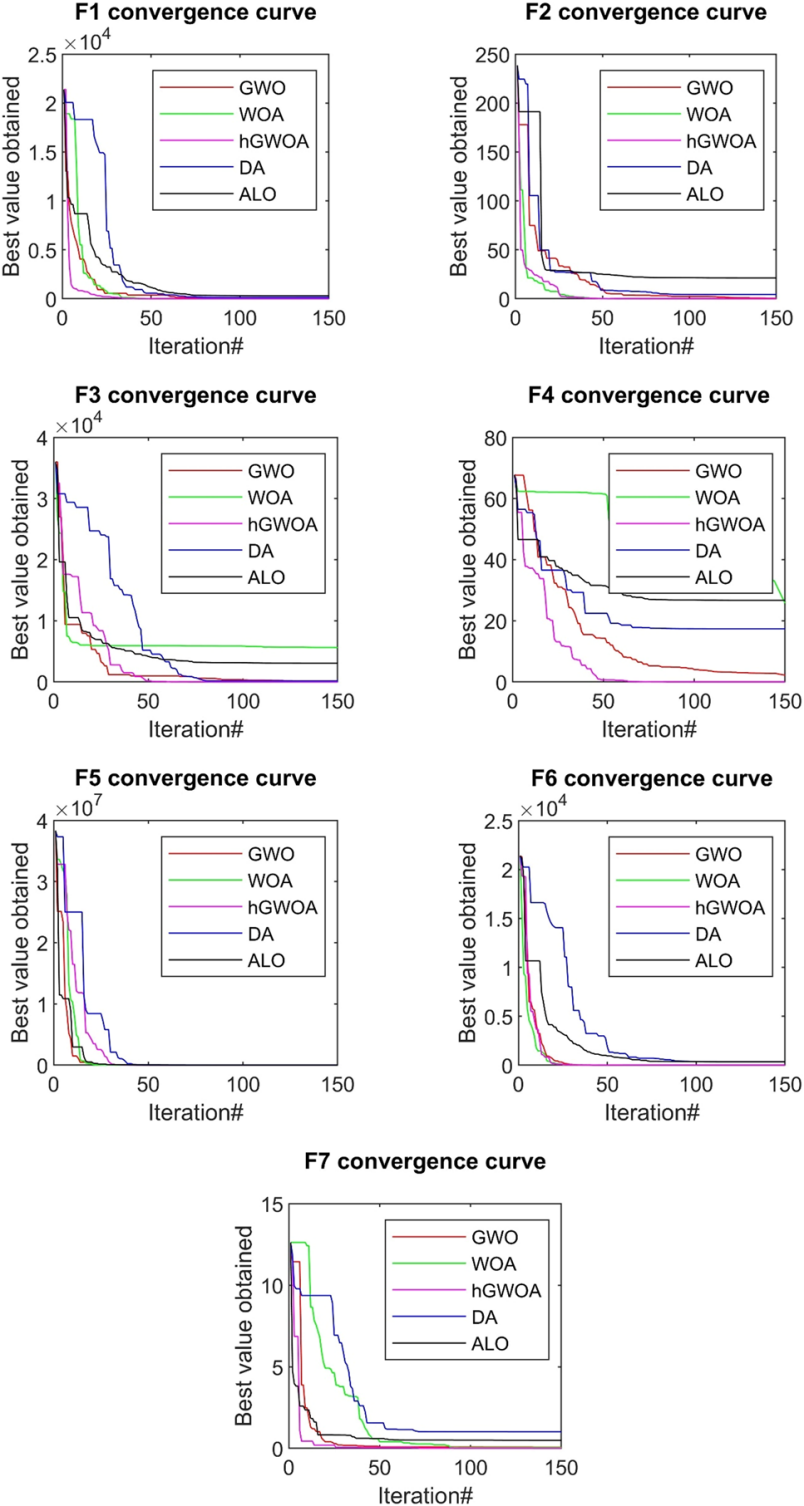
Convergence behavior of GWO, WOA, ALO, DA, and hGWOA for unimodal test functions.

**Figure 7:**
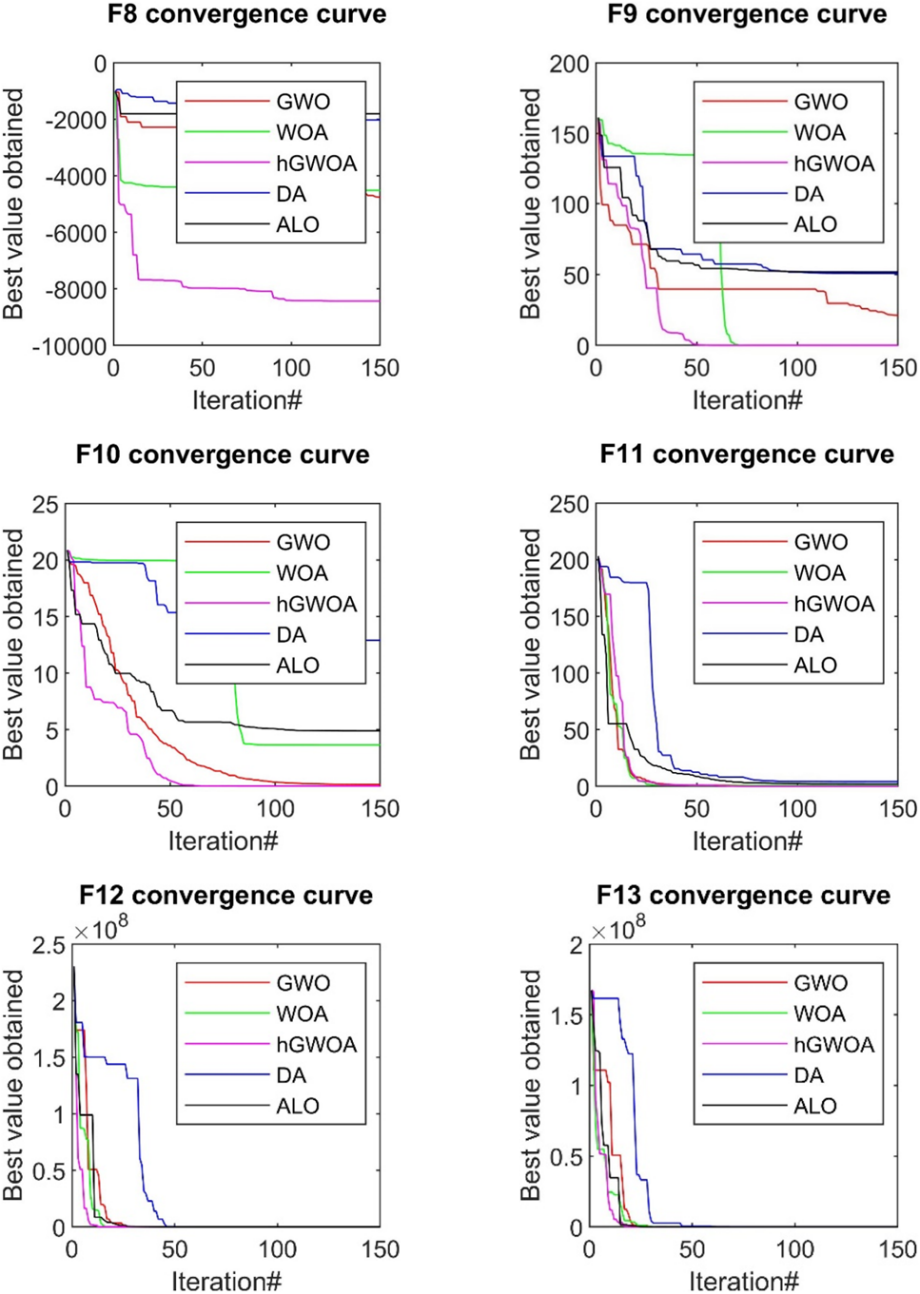
Convergence behavior of GWO, WOA, ALO, DA, and hGWOA for multi-modal functions.

**Figure 8:**
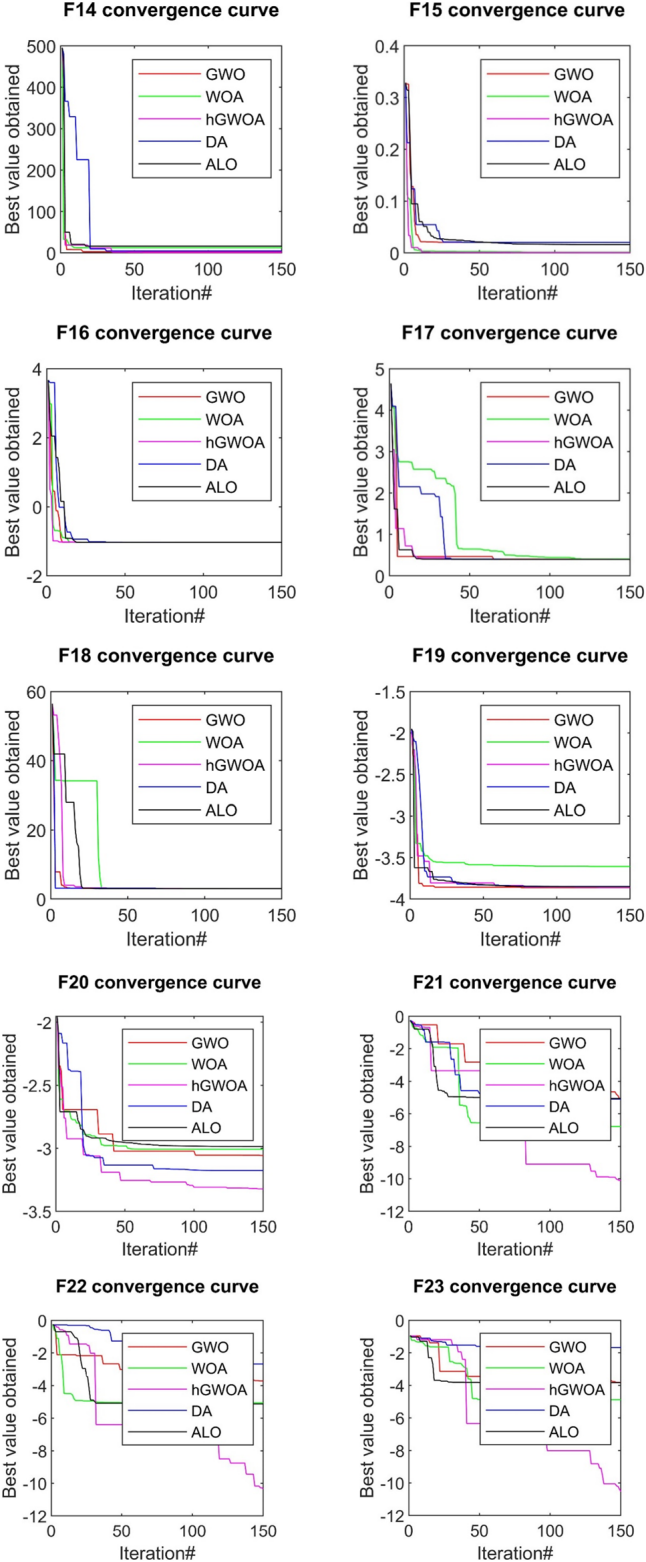
Convergence behavior of hGWOA, GWO, DA, ALO, WOA for composite functions.

The convergence analysis, which evaluates the efficacy of the hGWOA algorithm, is juxtaposed against other prominent algorithms, namely DA, ALO, WOA, and GWO. This comparative evaluation is visually represented in Figs. 6, 7, and 8. In this study, 30 exploration strategies were employed across 150 iterations, resulting in convergence diagrams. These diagrams vividly underscore the superior convergence aptitudes of hGWOA for a majority of the standard functions. Notably, the data suggests that hGWOA possesses a heightened probability of attaining optimal convergence compared to the other algorithms under examination.

### Convergence behaviours on CEC2017 benchmark function

The CEC2017 test functions form a suite of benchmark functions introduced during the 2017 IEEE Congress on Evolutionary Computation (CEC) competition, with an emphasis on real-parameter optimization. These benchmarks are highly esteemed within the evolutionary computation community and related fields. They serve as pivotal tools for evaluating and comparing the performance of optimization algorithms. Evolving from the benchmark collections of prior years, the CEC2017 suite has been rigorously designed to offer a wide array of challenges to optimization techniques.

Contrasted with the 23 traditional benchmark functions, the CEC2017 functions are viewed as more representative of realistic optimization scenarios. Their expansive coverage encompasses both unimodal and multi-modal landscapes, and they span separable as well as non-separable functions. Moreover, these functions feature shifted and rotated variants, providing an exhaustive testbed for algorithmic evaluations. Such a versatile set of testing scenarios allows researchers to evaluate the merits and limitations of various optimization algorithms across diverse contexts.

In this context, the efficacy of hGWOA has been assessed using the IEEE CEC2017 test suites, as referenced in^58^. These suites are broadly classified into four distinct categories: unimodal, multimodal, hybrid, and composition. Table 9 offers detailed definitions of the CEC2017 benchmark problems. To enhance the level of complexity and thoroughly evaluate the proposed method’s aptitude in addressing intricate optimization challenges, all functions within the CEC2017 suite have been configured to be 30-dimensional.

**Table 9:** CEC2017 benchmark functions.

Type	Function	Name	n	Range	fmin
Unimodal	*F*1	Shifted and Rotated Bent Cigar Function	30	[− 100, 100]	100
Unimodal	*F2*	Shifted and Rotated Zakharov Function	30	[− 100, 100]	200
Multimodal	*F3*	Shifted and Rotated Rosenbrock’s Function	30	[− 100, 100]	300
Multimodal	*F4*	Shifted and Rotated Rastrigin’s Function	30	[− 100, 100]	400
Multimodal	*F5*	Shifted and Rotated Expanded Scaffer’s *F7* Function	30	[− 100, 100]	500
Multimodal	*F6*	Shifted and Rotated Lunacek Bi_Rastrigin Function	30	[− 100, 100]	600
Multimodal	*F7*	Shifted and Rotated Non-Continuous Rastrigin’s Function	30	[− 100, 100]	700
Multimodal	*F8*	Shifted and Rotated Levy Function	30	[− 100, 100]	800
Multimodal	*F9*	Shifted and Rotated Schwefel’s Function	30	[− 100, 100]	900
Hybrid	*F*10	Hybrid Function 1 (*N* = 3)	30	[− 100, 100]	1000
Hybrid	*F*11	Hybrid Function 2 (*N* = 3)	30	[− 100, 100]	1100
Hybrid	*F*12	Hybrid Function 3 (*N* = 3)	30	[− 100, 100]	1200
Hybrid	*F*13	Hybrid Function 4 (*N* = 4)	30	[− 100, 100]	1300
Hybrid	*F*14	Hybrid Function 5 (*N* = 4)	30	[− 100, 100]	1400
Hybrid	*F*15	Hybrid Function 6 (*N* = 4)	30	[− 100, 100]	1500
Hybrid	*F*16	Hybrid Function 7 (*N* = 5)	30	[− 100, 100]	1600
Hybrid	*F*17	Hybrid Function 8 (*N* = 5)	30	[− 100, 100]	1700
Hybrid	*F*18	Hybrid Function 9 (*N* = 5)	30	[− 100, 100]	1800
Hybrid	*F19*	Hybrid Function 10 (*N* = 6)	30	[− 100, 100]	1900
Composition	*F*20	Composition Function 1 (*N* = 3)	30	[− 100, 100]	2000
Composition	*F*21	Composition Function 2 (*N* = 3)	30	[− 100, 100]	2100
Composition	*F*22	Composition Function 3 (*N* = 4)	30	[− 100, 100]	2200
Composition	*F*23	Composition Function 4 (*N* = 4)	30	[− 100, 100]	2300
Composition	*F*24	Composition Function 5 (*N* = 5)	30	[− 100, 100]	2400
Composition	*F*25	Composition Function 6 (*N* = 5)	30	[− 100, 100]	2500
Composition	*F*26	Composition Function 7 (*N* = 6)	30	[− 100, 100]	2600
Composition	*F*27	Composition Function 8 (*N* = 6)	30	[− 100, 100]	2700
Composition	*F*28	Composition Function 9 (*N* = 3)	30	[− 100, 100]	2800
Composition	*F29*	Composition Function 10 (*N* = 3)	30	[− 100, 100]	2900

For a comprehensive and unbiased evaluation, each algorithm was executed 30 times for every benchmark function. Following these runs, statistical analyses were carried out to evaluate both the central tendency and the dispersion of the data from these 30 trials. In the context of this study, 60 search agents were utilized, with each restricted to a maximum of 500 iterations. The results of the hGWOA approach are presented in Tables 10, juxtaposing its performance with that of other prominent algorithms such as DA, ALO, GWO, and WOA. A detailed examination of the data in Table 10 demonstrates that hGWOA consistently surpasses its nature-inspired peers, namely ALO, DA, WOA, and GWO, in the unimodal, multimodal, hybrid, and composition domains.

**Table 10:** Results of different algorithms on classical benchmark test functions.

Alg./Func.	hGWOA		GWO		WOA		DA		ALO	
	*Ave*	*Std*	*Ave*	*Std*	*Ave*	*Std*	*Ave*	*Std*	*Ave*	*Std*
***f***_***1***_	1.515E+08	6.713E+07	1.720E+09	8.722E+08	1.475E+10	5.256E+09	3.144E+10	3.144E+10	8.175E+03	5.323E+03
***f***_***2***_	1.097E+03	4.208E+02	3.880E+03	3.985E+03	3.864E+04	1.097E+03	6.167E+04	6.167E+04	5.627E+03	3.830E+03
***f***_***3***_	3.922E+02	2.526E+01	4.336E+02	5.024E+01	1.593E+03	3.922E+02	5.151E+03	5.151E+03	4.486E+02	5.209E+01
***f***_***4***_	9.315E+02	1.090E+02	1.499E+03	6.969E+02	2.492E+04	9.315E+02	3.856E+04	3.856E+04	7.350E+02	1.290E+02
***f***_***5***_	5.000E+02	1.288E−05	5.000E+02	7.565E−03	5.000E+02	5.000E+02	5.000E+02	5.000E+02	5.000E+02	1.762E−03
***f***_***6***_	8.457E+03	2.256E+03	1.898E+04	1.034E+04	1.171E+04	8.457E+03	1.840E+04	1.840E+04	5.323E+03	4.152E+03
***f***_***7***_	7.000E+02	2.291E−02	7.008E+02	6.523E−01	7.006E+02	7.000E+02	7.009E+02	7.009E+02	7.002E+02	1.663E−01
***f***_***8***_	8.011E+02	3.374E−01	8.029E+02	1.490E+00	8.241E+02	8.011E+02	8.262E+02	8.262E+02	8.091E+02	4.710E+00
***f***_***9***_	3.570E+03	3.551E+02	5.833E+03	2.075E+03	6.236E+03	3.570E+03	6.439E+03	6.439E+03	5.129E+03	6.655E+02
***f***_***10***_	9.568E+04	1.273E+04	1.271E+05	2.909E+04	2.295E+05	9.568E+04	1.901E+05	1.901E+05	1.141E+05	4.112E+04
***f***_***11***_	9.078E+06	4.968E+06	3.155E+07	2.287E+07	3.010E+08	9.078E+06	9.471E+08	9.471E+08	2.347E+07	1.887E+07
***f***_***12***_	9.239E+05	4.007E+05	3.497E+06	6.772E+06	1.181E+08	9.239E+05	1.427E+09	1.427E+09	1.577E+06	5.732E+05
***f***_***13***_	1.605E+05	3.738E+04	4.137E+05	3.760E+05	1.738E+06	1.605E+05	2.910E+05	2.910E+05	1.462E+06	1.562E+06
***f***_***14***_	1.622E+05	3.704E+04	4.534E+05	4.122E+05	3.363E+07	1.622E+05	1.492E+08	1.492E+08	3.904E+05	1.022E+05
***f***_***15***_	4.441E+04	1.133E+04	9.209E+05	1.349E+06	1.925E+06	4.441E+04	4.886E+05	4.886E+05	7.246E+04	5.953E+04
***f***_***16***_	4.149E+04	8.825E+03	6.391E+04	2.271E+04	7.823E+04	4.149E+04	5.645E+04	5.645E+04	4.391E+04	2.319E+04
***f***_***17***_	7.275E+04	1.301E+04	2.106E+05	3.155E+05	1.022E+05	7.275E+04	7.601E+04	7.601E+04	1.329E+05	4.026E+04
***f***_***18***_	7.048E+04	2.013E+04	1.714E+08	8.640E+08	1.293E+09	7.048E+04	7.308E+09	7.308E+09	7.753E+04	2.345E+04
***f***_***19***_	2.221E+03	1.296E+02	2.490E+03	2.389E+02	9.686E + 03	2.221E+03	7.480E+03	7.480E+03	5.070E+03	1.213E+03
***f***_***20***_	2.690E+03	1.600E+02	3.393E+03	6.967E+02	1.358E+04	2.690E+03	2.706E+04	2.706E+04	2.506E+03	3.148E+02
***f***_***21***_	2.279E+03	5.657E+00	2.291E+03	1.172E+01	3.610E+03	2.279E+03	3.857E+03	3.857E+03	2.440E+03	5.320E+01
***f***_***22***_	3.411E+03	3.658E+02	5.009E+03	1.623E+03	2.220E+04	3.411E+03	3.568E+04	3.568E+04	2.849E+03	9.544E+02
***f***_***23***_	2.872E+03	1.669E+02	3.794E+03	1.137E+03	1.795E+04	2.872E+03	2.434E+04	2.434E+04	2.517E+03	8.165E+01
***f***_***24***_	2.861E+03	1.255E+01	2.907E+03	3.988E+01	3.351E+03	2.861E+03	4.526E+03	4.526E+03	2.949E+03	5.562E+01
***f***_***25***_	3.355E+03	8.253E+00	3.388E+03	3.446E+01	4.213E+03	3.355E+03	4.085E+03	4.085E+03	3.439E+03	6.695E+01
***f***_***26***_	3.121E+03	5.662E+00	3.133E+03	1.672E+01	3.713E+03	3.121E+03	3.418E+03	3.418E+03	3.294E+03	7.807E+01
***f***_***27***_	3.092E+03	5.654E+01	3.144E+03	6.524E+01	3.498E+03	3.092E+03	4.296E+03	4.296E+03	3.152E+03	7.216E+01
***f***_***28***_	1.653E+05	9.541E+04	1.642E+07	2.428E+07	3.969E+08	1.653E+05	4.643E+08	4.643E+08	2.785E+06	6.781E+06
***f***_***29***_	4.587E+05	2.153E+05	2.016E+07	3.351E+07	5.015E+08	4.587E+05	2.829E+09	2.829E+09	3.876E+07	7.734E+07

### Different versions of the CVRP.

For the TSP as delineated in references^55,59^, the computational complexity is recognized to escalate exponentially with the augmentation in the number of cities. To elucidate, a TSP encompassing *n* cities entails considering 1/2*(n−1)! feasible routes. Taking an illustrative example where *n* = *16*, the total number of potential routes amounts to an overwhelming 6.54 × 10^11^. This vast array of route permutations renders the TSP exceptionally computation-intensive. In light of this, when considering the VRP, which essentially comprises multiple intertwined TSPs, the computational complexity is magnified substantially.

### Case study 1

In the first case study addressing the CVRP challenge, the setting encompasses a central warehouse tasked with catering to eight distinct customers. This operation is facilitated by two delivery trucks, each possessing a capacity to transport eight vehicles. The Euclidean distances, along with the specific delivery requirements pertinent to each customer, are tabulated in Table 11. The primary objective of this case study revolves around minimizing the cumulative distance traversed by the two delivery trucks, ensuring that all constraints intrinsic to the VRP are met in the process.

**Table 11:** Customer Euclidean distance and delivery requirements of 8-customer problem^60^.

Node	0	1	2	3	4	5	6	7	8	Demand
0	0	4	6	7.5	9	20	10	16	8	
1	4	0	6.5	4	10	5	7.5	11	10	1
2	6	6.5	0	7.5	10	10	7.5	7.5	7.5	2
3	7.5	4	7.5	0	10	5	9	9	15	1
4	9	10	10	10	0	10	7.5	7.5	10	2
5	20	5	10	5	10	0	7	9	7.5	1
6	10	7.5	7.5	9	7.5	7	0	7	10	4
7	16	11	7.5	6	7.5	9	7	0	10	2
8	8	10	7.5	15	10	7.5	10	10	0	2

Table 12 delineates the results derived from a diverse array of algorithms applied to the given problem. This includes methodologies as proposed in reference^60^, complemented by outcomes from distinct algorithms like DA, GWO, ALO, and WOA. Notably, the mean percentage deviation (*%dev*) for the hGWOA stands out as superior. It registers a more favorable performance than WOA (0.44%), DA (1.51%), ALO (2.14%), GWO (1.44%), MHPSO (1.74%), DPGA (2.73%), and SGA (4.03%).

**Table 12:** Results of different algorithms on 8-customer problem.

Algorithm	Scattering of optimal solutions					Max	Min	Mean	Deviation ratio of the mean best solution
hGWOA	67.5	68	67.5	67.5	67.5	68	67.5	67.700	0.00%
	68	67.5	67.5	67.5	67.5				
	68	68	68	68	68				
	68	67.5	67.5	67.5	67.5				
WOA	68.5	68	69	68	68	69	67.5	68.000	0.44%
	68	67.5	67.5	67.5	68				
	69	68	67.5	68.5	67.5				
	68	68.5	68	67.5	67.5				
DA	71.5	67.5	71.5	68	67.5	71.5	67.5	68.725	1.51%
	69	70	70.5	68	69				
	70	67.5	67.5	69	68				
	67.5	68	68	67.5	69				
ALO	71.5	68	71.5	68	67.5	71.5	67.5	69.150	2.14%
	69	70	70.5	68	69				
	70	68	71	69	68				
	71.5	68	68	67.5	69				
MHPSO^60^	69.5	67.5	69	69	70	70	67.5	68.875	1.74%
	69.5	70	69	67.5	67.5				
	69	69.5	69	70	67.5				
	70	69	67.5	70	67.5				
GWO	70	68.5	69	69	69	70	67.5	68.675	1.44%
	68	69	67.5	70	68.5				
	68.5	68.5	67.5	68	69.5				
	68	67.5	69.5	68.5	69.5				
DPGA^60^	70	69	67.5	71	69	72	67.5	69.550	2.73%
	70.5	72	67.5	71.5	69				
	67.5	69	71	70	67.5				
	70.5	69	69.5	71	69				
SGA^60^	69	72	73.5	69	70	75.5	67.5	70.425	4.03%
	71	67.5	69	69	75.5				
	70	69.5	69	73	69				
	74	70	69.5	69	70				

**Figure 9:**
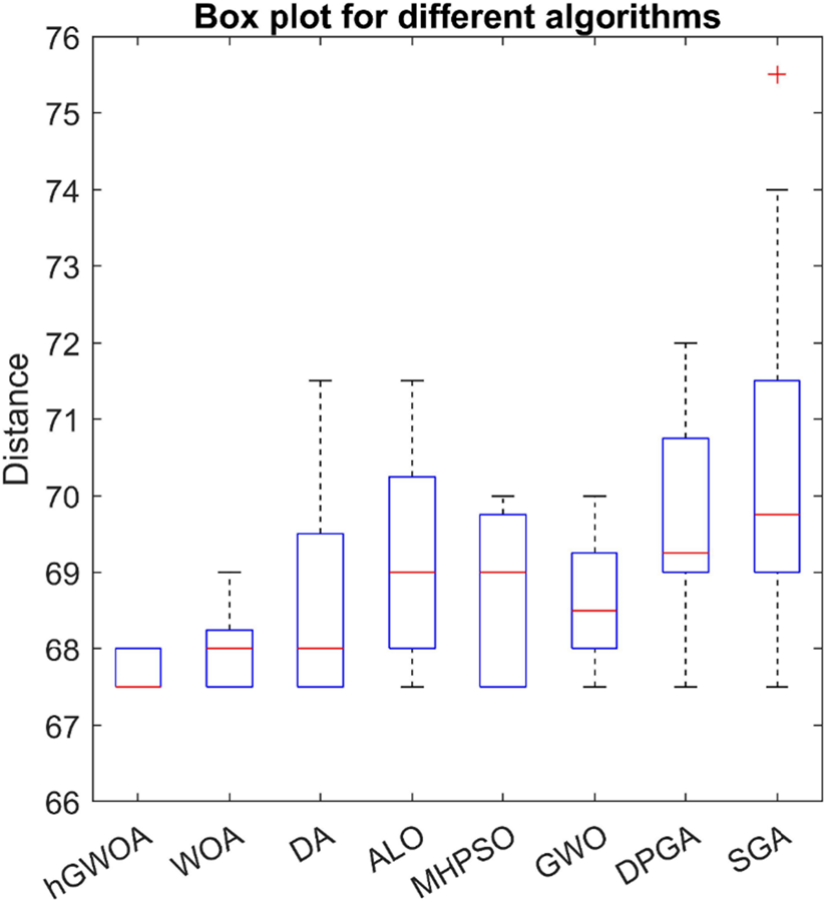
Boxplot of hGWOA, WOA, DA, ALO, MHPSO, DPGA, SGA and GWO on 8-customer problem.

**Figure 10:**
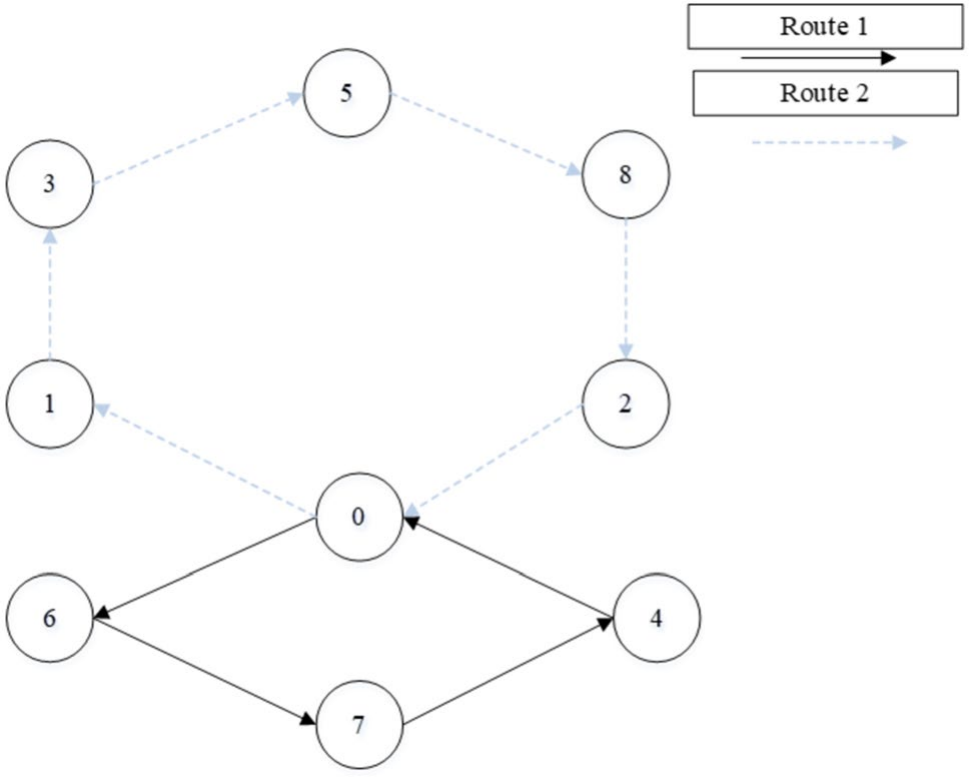
Best solution for the CVRP of 8-customer problem.

While all considered algorithms yielded commendable results, the average outcomes from hGWOA surpassed the rest, underscoring its superior stability in both the mining and exploration phases. Complementing these observations, Fig. 9 visually portrays hGWOA's advantageous data distribution relative to its counterparts. Among the results, the pinnacle solution achieved a commendable total distance of 67.5 units. Leveraging the hGWOA algorithm, the navigation routes for the two vehicles were computed, the details of which are tabulated in Table 13. A more granular graphical representation of these routes can be viewed in Fig. 10.

**Table 13:** Routing of vehicles and distance using hGWOA algorithm on 8-customer problem.

Routes of the vehicles on 8-customer problem		Distance
Route 1	0- > Customer 6- > Customer 7- > Customer 4- > 0	33.5
Route 2	0- > Customer 1- > Customer 3- > Customer 5- > Customer 8- > Customer 2- > 0	34
Total distance: 67.5 units

For implementation, the chosen algorithms were rendered in Java. Subsequent integrations and tests were conducted on a personal computer equipped with an Intel(R) Core(TM) Processor I7-1165G7 clocked at 2.80 GHz. Each algorithm underwent 20 runs, employing 20 distinct exploration strategies and encapsulating 50 iterations for all the CVRP scenarios.

### Case study 2

In the second case study addressing the CVRP problem, the intrinsic complexity of the TSP issue is addressed by leveraging data from Azad’s^40^ study. This research focuses on a hub-and-spoke delivery system serving 25 cement customers. A pioneering approach to the CVRP is proposed, implementing a genetic algorithm technique and deploying a fleet of five delivery trucks, each having a capacity of 1500 bags. Using the coordinate data provided in Table 14, we derive matrices that represent distances among customers and their specific demands. These matrices are presented in Tables 15 and 16, respectively. The primary aim is to optimize delivery routes for the 25 customers with a fleet of five trucks, thereby minimizing the total distance traveled while still adhering to the fundamental constraints inherent in the CVRP.

**Table 14:** The coordinates of 25 customers and their respective demands per customer^40^.

Customer no.	X	Y	Demand
1	0.00	0.00	0.00
2	24.830	21.72	1000.0
3	32.610	15.00	400.0
4	53.120	21.720	150.0
5	26.190	− 17.850	50.0
6	3.002540	− 9.852650	300.0
7	9.9800730	13.76220	100.0
8	13.4230	12.5850	200.0
9	14.2290	15.44455	50
10	2.343654	15.92851	250
11	8.081236	14.50013	75
12	12.45585	− 11.2311	250
13	5.425	14.412	500
14	25.312	46.223	150
15	21.312	46.223	50
16	5.6	10.044	200
17	32.01	− 38.67	300
18	− 0.131	− 38.67	150
19	20.02	6.315	250
20	− 39.5217	− 38.67	300
21	19.49	23.456	350
22	56	-43.97	150
23	36	45	180
24	39	26.408	350
25	26.905	26.4082	250
26	15	15.95	275

**Table 15:** Customer Euclidean distance and delivery requirements of 25-customer problem.

Node	1	2	3	4	5	6	7	8	9	10	11	12	Demand
1	0	33.0	35.9	57.4	31.7	10.3	17.0	18.4	21.0	16.1	16.6	16.8	1000
2	33.0	0	10.3	28.3	39.6	38.4	16.8	14.6	12.3	23.2	18.2	35.2	400
3	35.9	10.3	0	21.6	33.5	38.7	22.7	19.3	18.4	30.3	24.5	33.1	150
4	57.4	28.3	21.6	0	47.9	59.2	43.9	40.7	39.4	51.1	45.6	52.3	50
5	31.7	39.6	33.5	47.9	0	24.5	35.5	33.0	35.4	41.3	37.1	15.2	300
6	10.3	38.4	38.7	59.2	24.5	0	24.6	24.7	27.7	25.8	24.9	9.6	100
7	17.0	16.8	22.7	43.9	35.5	24.6	0	3.6	4.6	7.9	2.0	25.1	200
8	18.4	14.6	19.3	40.7	33.0	24.7	3.6	0	3.0	11.6	5.7	23.8	50
9	21.0	12.3	18.4	39.4	35.4	27.7	4.6	3.0	0	11.9	6.2	26.7	250
10	16.1	23.2	30.3	51.1	41.3	25.8	7.9	11.6	11.9	0	5.9	29.0	75
11	16.6	18.2	24.5	45.6	37.1	24.9	2.0	5.7	6.2	5.9	0	26.1	250
12	16.8	35.2	33.1	52.3	15.2	9.6	25.1	23.8	26.7	29.0	26.1	0	500
13	15.4	20.7	27.2	48.3	38.4	24.4	4.6	8.2	8.9	3.4	2.7	26.6	150
14	52.7	24.5	32.1	37.1	64.1	60.4	35.9	35.7	32.7	38.0	36.1	58.9	50
15	50.9	24.8	33.2	40.2	64.3	59.0	34.4	34.6	31.6	35.7	34.4	58.1	200
16	11.5	22.5	27.5	48.9	34.7	20.1	5.8	8.2	10.2	6.7	5.1	22.4	300
17	50.2	60.8	53.7	64.0	21.6	40.9	56.9	54.5	57.0	62.1	58.3	33.7	150
18	38.7	65.3	62.9	80.5	33.6	29.0	53.4	53.0	56.0	54.7	53.8	30.2	250
19	21.0	16.1	15.3	36.5	24.9	23.5	12.5	9.1	10.8	20.1	14.5	19.1	300
20	55.3	88.3	89.9	111.0	68.9	51.4	72.1	73.7	76.3	68.8	71.4	58.8	350
21	30.5	5.6	15.6	33.7	41.8	37.2	13.6	12.4	9.6	18.7	14.5	35.4	150
22	71.2	72.7	63.4	65.8	39.6	63.0	73.8	70.8	72.6	80.4	75.6	54.5	180
23	57.6	25.8	30.2	28.9	63.6	64.0	40.7	39.5	36.7	44.5	41.3	61.0	350
24	47.1	14.9	13.1	14.9	46.1	51.1	31.7	29.1	27.1	38.1	33.1	46.1	250
25	37.7	5.1	12.8	26.6	44.3	43.4	21.1	19.3	16.8	26.7	22.3	40.3	275
26	21.9	11.4	17.6	38.6	35.6	28.5	5.5	3.7	0.9	12.7	7.1	27.3	1000

**Table 16:** Customer Euclidean distance and delivery requirements of 25-customer problem (continued).

Node	13	14	15	16	17	18	19	20	21	22	23	24	25	26	Demand
1	15.4	52.7	50.9	11.5	50.2	38.7	21.0	55.3	30.5	71.2	57.6	47.1	37.7	21.9	1000
2	20.7	24.5	24.8	22.5	60.8	65.3	16.1	88.3	5.6	72.7	25.8	14.9	5.1	11.4	400
3	27.2	32.1	33.2	27.5	53.7	62.9	15.3	89.9	15.6	63.4	30.2	13.1	12.8	17.6	150
4	48.3	37.1	40.2	48.9	64.0	80.5	36.5	111.0	33.7	65.8	28.9	14.9	26.6	38.6	50
5	38.4	64.1	64.3	34.7	21.6	33.6	24.9	68.9	41.8	39.6	63.6	46.1	44.3	35.6	300
6	24.4	60.4	59.0	20.1	40.9	29.0	23.5	51.4	37.2	63.0	64.0	51.1	43.4	28.5	100
7	4.6	35.9	34.4	5.8	56.9	53.4	12.5	72.1	13.6	73.8	40.7	31.7	21.1	5.5	200
8	8.2	35.7	34.6	8.2	54.5	53.0	9.1	73.7	12.4	70.8	39.5	29.1	19.3	3.7	50
9	8.9	32.7	31.6	10.2	57.0	56.0	10.8	76.3	9.6	72.6	36.7	27.1	16.8	0.9	250
10	3.4	38.0	35.7	6.7	62.1	54.7	20.1	68.8	18.7	80.4	44.5	38.1	26.7	12.7	75
11	2.7	36.1	34.4	5.1	58.3	53.8	14.5	71.4	14.5	75.6	41.3	33.1	22.3	7.1	250
12	26.6	58.9	58.1	22.4	33.7	30.2	19.1	58.8	35.4	54.5	61.0	46.1	40.3	27.3	500
13	0	37.5	35.6	4.4	59.4	53.4	16.7	69.6	16.7	77.2	43.2	35.7	24.6	9.7	150
14	37.5	0	4.0	41.2	85.2	88.6	40.3	107.0	23.5	95.3	10.8	24.1	19.9	32.0	50
15	35.6	4.0	0	39.4	85.6	87.6	39.9	104.0	22.8	96.6	14.7	26.6	20.6	30.9	200
16	4.4	41.2	39.4	0	55.4	49.0	14.9	66.4	19.3	73.9	46.3	37.2	26.9	11.1	300
17	59.4	85.2	85.6	55.4	0	32.1	46.6	71.5	63.4	24.6	83.8	65.5	65.3	57.2	150
18	53.4	88.6	87.6	49.0	32.1	0	49.3	39.4	65.2	56.4	91.1	75.9	70.5	56.7	250
19	16.7	40.3	39.9	14.9	46.6	49.3	0	74.6	17.1	61.8	41.9	27.6	21.2	10.9	300
20	69.6	107.0	104.0	66.4	71.5	39.4	74.6	0	85.7	95.7	113.0	102.0	93.0	77.2	350
21	16.7	23.5	22.8	19.3	63.4	65.2	17.1	85.7	0	76.7	27.1	19.7	8.0	8.8	150
22	77.2	95.3	96.6	73.9	24.6	56.4	61.8	95.7	76.7	0	91.2	72.4	76.2	72.6	180
23	43.2	10.8	14.7	46.3	83.8	91.1	41.9	113.0	27.1	91.2	0	18.8	20.7	35.8	350
24	35.7	24.1	26.6	37.2	65.5	75.9	27.6	102.0	19.7	72.4	18.8	0	12.1	26.2	250
25	24.6	19.9	20.6	26.9	65.3	70.5	21.2	93.0	8.0	76.2	20.7	12.1	0	15.8	275
26	9.7	32.0	30.9	11.1	57.2	56.7	10.9	77.2	8.8	72.6	35.8	26.2	15.8	0	1000

**Figure 11:**
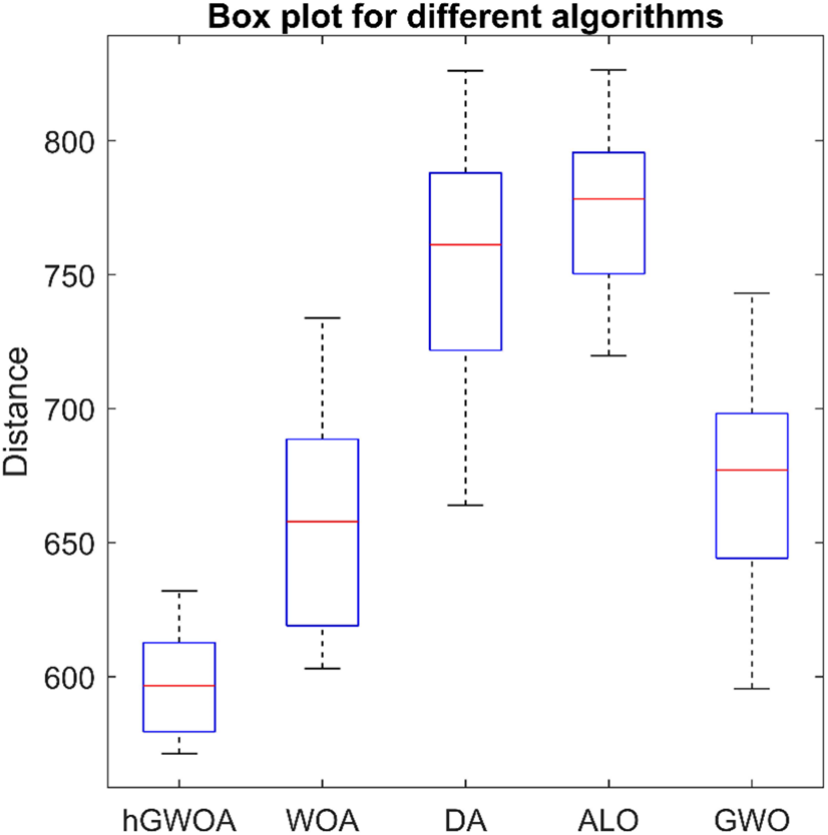
Boxplot of hGWOA, WOA, DA, ALO and GWO on 25-customer problem.

**Figure 12:**
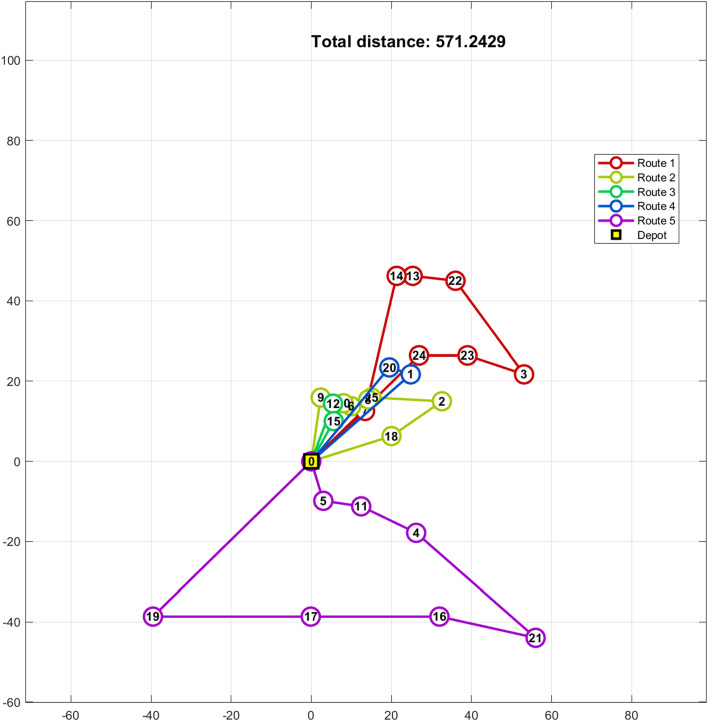
Best solution for the CVRP of 25-customer problem.

Table 17 presents the results from various algorithm implementations. Notably, the *%dev* best solution achieved by the hGWOA algorithm surpasses that of other optimization techniques. It outperforms WOA by 6%, DA by 16%, ALO by 26%, GWO by 4%, and GA by 31%. Moreover, Fig. 11 provides a visual representation that highlights the superior data distribution of hGWOA compared to other algorithms. This study's findings accentuate the efficacy of the hGWOA algorithm in obtaining the optimal solution, with a total distance of 571.24 units. Table 18 details the delivery routes for the five trucks as determined by hGWOA, and a graphical representation is provided in Fig. 12.

**Table 17:** Results of different algorithms on 25-customer problem.

Algorithm	Scattering of optimal solutions					Max	Min	Mean	Deviation ratio of the best solution
hGWOA	594.17	574.85	628.94	611.93	613.86	632.01	571.24	596.62	0%
	578.03	574.85	613.37	631.29	579.05				
	579.81	594.89	594.66	571.24	600.56				
	586.41	602.19	632.01	598.34	604.63				
WOA	658.15	612.47	673.42	649.31	657.48	733.84	603.02	657.82	6%
	656.99	606.54	615.09	683.35	603.02				
	700.35	733.84	636.72	622.83	697.92				
	687.39	689.73	706.03	606.32	669.25				
DA	796.06	672.63	748.23	826.09	704.53	826.09	663.92	761.16	16%
	802.63	788.09	663.92	762.49	735.05				
	784.75	785.36	760.21	740.96	787.83				
	708.49	796.06	762.10	743.28	688.35				
ALO	743.07	768.87	752.43	725.64	796.06	826.37	719.77	778.24	26%
	746.15	784.73	795.13	719.77	754.84				
	786.55	826.37	750.95	819.91	772.41				
	816.34	784.41	798.52	749.69	784.07				
GWO	743.07	679.59	630.99	712.28	638.03	743.07	595.48	677.07	4%
	648.24	689.98	663.92	640.05	674.55				
	656.40	684.28	639.23	684.51	651.81				
	721.52	702.27	714.97	595.48	694.11				
GA^40^	NA	NA	NA	NA	NA	892.47	747.70	NA	31%

**Table 18:** Routing of vehicles and distance using hGWOA algorithm on 25-customer problem.

Routes of the vehicles on 25-customer problem		Distance
Route 1	0- > Customer 24- > Customer 23- > Customer 3- > Customer 22- > Customer 13- > Customer 14- > Customer 7- > 0	161.28
Route 2	0- > Customer 18- > Customer 2- > Customer 25- > Customer 8- > Customer 6- > Customer 10- > Customer 9- > 0	83.46
Route 3	0- > Customer 12- > Customer 15- > 0	31.27
Route 4	0- > Customer 1- > Customer 20- > 0	69.1
Route 5	0- > Customer 19- > Customer 17- > Customer 16- > Customer 21- > Customer 4- > Customer 11- > Customer 5- > 0	226.13
Total distance: 571.24 units

For experimental implementation, the algorithms were coded in Java and executed on a personal computer powered by an Intel(R) Core(TM) i7-1165G7 processor with a clock speed of 2.80 GHz. Each algorithm was tested over 20 runs, employing 60 search agents, for a total of 200 iterations in all CVRP scenarios.

### Real CVRP in Viet Nam

In the real case study addressing the CVRP issue, authentic delivery data from a cement supplier servicing 30 customers was scrutinized within a shaft-and-spokes distribution framework. This data was pivotal in tackling the intricacies associated with the TSP challenge. The supplier operated with a fleet of six delivery trucks, each with a capacity of 700 bags. Utilizing the given distance coordinates, we derived a distance matrix for each customer, outlining their specific demands, as illustrated in Tables 19 and 20. The primary goal was to efficiently cater to all 30 customers using the six trucks, minimizing the total travel distance, all while adhering to the parameters set by the CVRP.

**Table 19:** Customer Euclidean distance and delivery requirements of 30-customer problem.

Node	0	1	2	3	4	5	6	7	8	9	10	11	12	13	14	15	16	Demand
0	0	67.2	53.9	33.5	15.0	50.3	51.0	46.0	40.5	12.5	47.9	61.1	24.6	52.5	42.0	43.4	45.2	0
1	67.2	0	121.1	34.6	64.5	115.8	95.9	73.6	48.0	57.6	50.0	84.9	91.0	70.4	31.4	110.3	55.4	170
2	53.9	121.1	0	87.0	60.2	17.4	61.2	76.8	87.6	64.2	92.7	85.8	31.1	86.1	94.2	12.9	87.6	40
3	33.5	34.6	87.0	0	34.7	81.2	65.2	46.6	34.1	23.1	30.4	61.0	56.6	47.2	11.3	75.9	32.6	50
4	15.0	64.5	60.2	34.7	0	60.5	66.0	59.6	28.1	22.4	57.1	75.1	35.3	65.2	45.4	52.0	55.7	100
5	50.3	115.8	17.4	81.2	60.5	0	44.8	62.9	88.6	58.2	81.5	70.2	25.7	72.2	86.7	10.5	75.9	20
6	51.0	96.0	61.2	65.2	66.0	44.8	0	24.8	88.9	48.5	49.3	26.2	42.0	32.7	64.6	49.3	43.1	130
7	46.0	73.6	76.8	46.6	59.6	62.9	24.8	0	76.2	37.8	24.9	15.6	49.8	9.3	43.0	64.0	18.8	190
8	40.5	48.0	87.6	34.1	28.1	88.6	88.9	76.2	0	40.5	64.3	91.6	63.3	79.0	44.5	80.0	65.6	170
9	12.5	57.6	64.2	23.1	22.4	58.2	48.5	37.8	40.5	0	35.8	53.3	33.5	42.8	30.2	52.9	33.6	50
10	47.9	50.0	92.7	30.4	57.1	81.5	49.3	24.9	64.3	35.8	0	34.9	62.3	20.4	22.2	80.0	6.3	120
11	61.1	84.9	85.8	61.0	75.1	70.2	26.2	15.6	91.6	53.3	34.9	0	61.8	14.5	55.8	73.3	29.8	70
12	24.6	91.0	31.1	56.6	35.3	25.7	42.0	49.8	63.3	33.5	62.3	61.8	0	58.6	63.2	19.3	57.7	140
13	52.5	70.4	86.1	47.2	65.2	72.2	32.7	9.3	79.0	42.8	20.4	14.5	58.6	0	41.4	73.3	15.4	70
14	42.0	31.4	94.2	11.3	45.4	86.7	64.6	43.0	44.5	30.2	22.2	55.8	63.2	41.4	0	82.4	26.1	90
15	43.4	110.3	12.9	75.9	52.0	10.5	49.3	64.0	80.0	52.9	80.0	73.3	19.3	73.3	82.4	0	74.8	100
16	45.2	55.4	87.6	32.6	55.7	75.9	43.1	18.8	65.6	33.6	6.3	29.8	57.7	15.4	26.1	74.8	0	170
17	56.9	10.4	110.7	24.6	54.1	105.7	87.8	66.5	39.4	47.5	44.3	78.9	80.8	64.3	23.5	100.0	49.0	200
18	54.0	43.4	101.0	30.7	61.5	90.3	58.4	33.9	64.6	41.5	9.2	42.3	70.3	28.3	20.3	88.4	15.5	180
19	36.1	35.3	88.8	17.1	29.8	86.3	78.2	62.2	17.6	30.7	47.5	77.1	60.6	63.7	27.0	79.2	49.5	140
20	29.0	47.5	78.7	15.9	37.0	70.3	49.5	30.9	46.5	16.5	20.2	45.7	47.6	32.6	16.6	66.6	19.6	80
21	53.0	26.7	104.8	21.7	56.3	96.5	70.9	47.6	52.5	41.0	23.4	58.3	73.7	43.8	11.1	92.8	29.0	200
22	33.6	64.0	72.3	34.1	46.5	60.6	32.0	13.7	62.6	24.3	20.9	29.3	42.6	18.9	32.6	59.5	15.4	120
23	44.5	70.9	78.3	53.1	29.5	83.7	95.5	88.2	22.9	50.5	81.5	103.8	61.1	92.9	64.3	73.8	81.5	80
24	34.1	86.4	51.8	59.0	24.5	59.1	80.0	79.6	42.0	45.2	80.9	94.4	40.0	86.5	69.9	48.8	78.8	190
25	36.6	94.1	43.6	65.0	30.4	52.5	78.0	80.5	50.9	48.7	84.5	94.6	36.4	88.0	75.5	41.9	81.8	170
26	3.6	69.5	51.7	35.3	18.4	47.3	47.7	43.7	44.0	13.1	47.4	58.7	21.7	50.7	43.2	40.9	44.3	80
27	57.6	84.6	81.6	59.3	71.8	66.1	22.4	12.9	89.2	50.3	34.7	4.2	57.6	14.4	54.9	69.1	29.2	130
28	46.0	110.4	23.2	75.9	57.4	6.9	38.2	56.0	85.4	53.0	74.9	63.4	22.1	65.4	80.8	13.0	69.3	170
29	15.7	82.4	39.2	49.0	21.9	38.7	53.0	55.0	50.0	27.7	61.8	68.9	14.4	62.8	57.6	30.2	58.2	60
30	43.2	87.3	62.2	63.5	31.0	70.5	91.1	89.2	40.2	53.2	88.1	104.2	51.6	95.5	74.7	60.1	86.7	160

**Table 20:** Customer Euclidean distance and delivery requirements of 30-customer problem (continued).

Node	17	18	19	20	21	22	23	24	25	26	27	28	29	30	Demand
0	56.9	54.0	36.1	29.0	53.0	33.6	44.5	34.1	36.6	3.6	57.6	46.0	15.7	43.2	0
1	10.4	43.4	35.3	47.5	26.7	64.0	70.9	86.4	94.1	69.5	84.6	110.4	82.4	87.3	170
2	110.7	101.0	88.8	78.7	104.8	72.3	78.3	51.8	43.6	51.7	81.6	23.2	39.2	62.2	40
3	24.6	30.7	17.1	15.9	21.7	34.1	53.1	59.0	65.0	35.3	59.3	75.9	49.0	63.5	50
4	54.1	61.5	29.8	37.0	56.3	46.5	29.5	24.5	30.4	18.4	71.8	57.4	21.9	31.0	100
5	105.7	90.3	86.3	70.3	96.5	60.6	83.7	59.1	52.5	47.3	66.1	6.9	38.7	70.5	20
6	87.8	58.4	78.2	49.5	70.9	32.0	95.5	80.0	78.0	47.7	22.4	38.2	53.0	91.1	130
7	66.5	33.9	62.2	30.9	47.6	13.7	88.2	79.6	80.5	43.7	12.9	56.0	55.0	89.2	190
8	39.4	64.6	17.6	46.5	52.5	62.6	22.9	42.0	50.9	44.0	89.2	85.4	50.0	40.2	170
9	47.5	41.5	30.7	16.5	41.0	24.3	50.5	45.2	48.7	13.1	50.3	53.0	27.7	53.2	50
10	44.3	9.2	47.5	20.2	23.4	20.9	81.5	80.9	84.5	47.4	34.7	74.9	61.8	88.1	120
11	78.9	42.3	77.1	45.7	58.3	29.3	103.8	94.4	94.6	58.7	4.2	63.4	68.9	104.2	70
12	80.8	70.3	60.6	47.6	73.7	42.6	61.1	40.0	36.4	21.7	57.6	22.1	14.4	51.6	140
13	64.3	28.3	63.7	32.6	43.8	18.9	92.9	86.5	88.0	50.7	14.4	65.4	62.8	95.5	70
14	23.5	20.3	27.0	16.6	11.1	32.6	64.3	69.9	75.5	43.2	54.9	80.8	57.6	74.7	90
15	100.0	88.4	79.2	66.6	92.8	59.5	73.8	48.8	41.9	40.9	69.1	13.0	30.2	60.1	100
16	49.0	15.5	49.5	19.6	29.0	15.4	81.5	78.8	81.8	44.3	29.2	69.3	58.2	86.7	170
17	0	39.1	25.3	38.6	22.1	55.8	62.3	76.4	83.9	59.2	78.1	100.5	72.0	77.8	200
18	39.1	0	47.2	25.1	17.1	29.9	83.6	85.7	90.1	54.0	42.7	83.8	68.6	92.1	180
19	25.3	47.2	0	31.4	35.0	48.9	38.8	51.2	58.8	39.1	75.1	81.9	49.7	52.8	140
20	38.6	25.1	31.4	0	26.2	18.1	61.9	61.0	65.0	29.3	43.7	64.4	44.0	67.9	80
21	22.1	17.1	35.0	26.2	0	39.5	73.5	80.7	86.5	54.1	58.1	90.5	68.6	85.1	200
22	55.8	29.9	48.9	18.1	39.5	0	74.7	67.7	69.4	31.9	26.6	54.0	44.7	76.7	120
23	62.3	83.6	38.8	61.9	73.5	74.7	0	26.9	36.0	47.8	100.7	82.2	46.7	20.1	80
24	76.4	85.7	51.2	61.0	80.7	67.7	26.9	0	9.2	35.9	90.6	58.8	27.1	11.7	190
25	83.9	90.1	58.8	65.0	86.5	69.4	36.0	9.2	0	37.6	90.7	53.0	25.7	18.6	170
26	59.2	54.0	39.1	29.3	54.1	31.9	47.8	35.9	37.6	0	55.1	42.8	14.7	45.6	80
27	78.1	42.7	75.1	43.7	58.1	26.6	100.7	90.6	90.7	55.1	0	59.3	64.9	100.6	130
28	100.5	83.8	81.9	64.4	90.5	54.0	82.2	58.8	53.0	42.8	59.3	0	36.1	70.4	170
29	72.0	68.6	49.7	44.0	68.6	44.7	46.7	27.1	25.7	14.7	64.9	36.1	0	38.3	60
30	77.8	92.1	52.8	67.9	85.1	76.7	20.1	11.7	18.6	45.6	100.6	70.4	38.3	0	160

**Figure 13:**
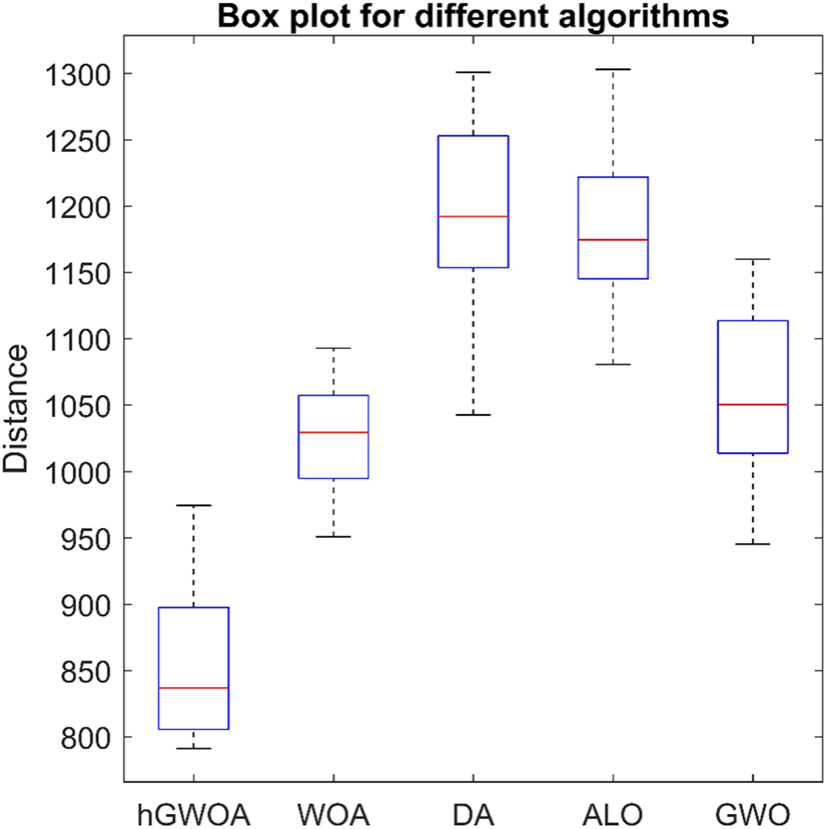
Box plot of hGWOA, WOA, DA, ALO and GWO on 30-customer problem.

**Figure 14:**
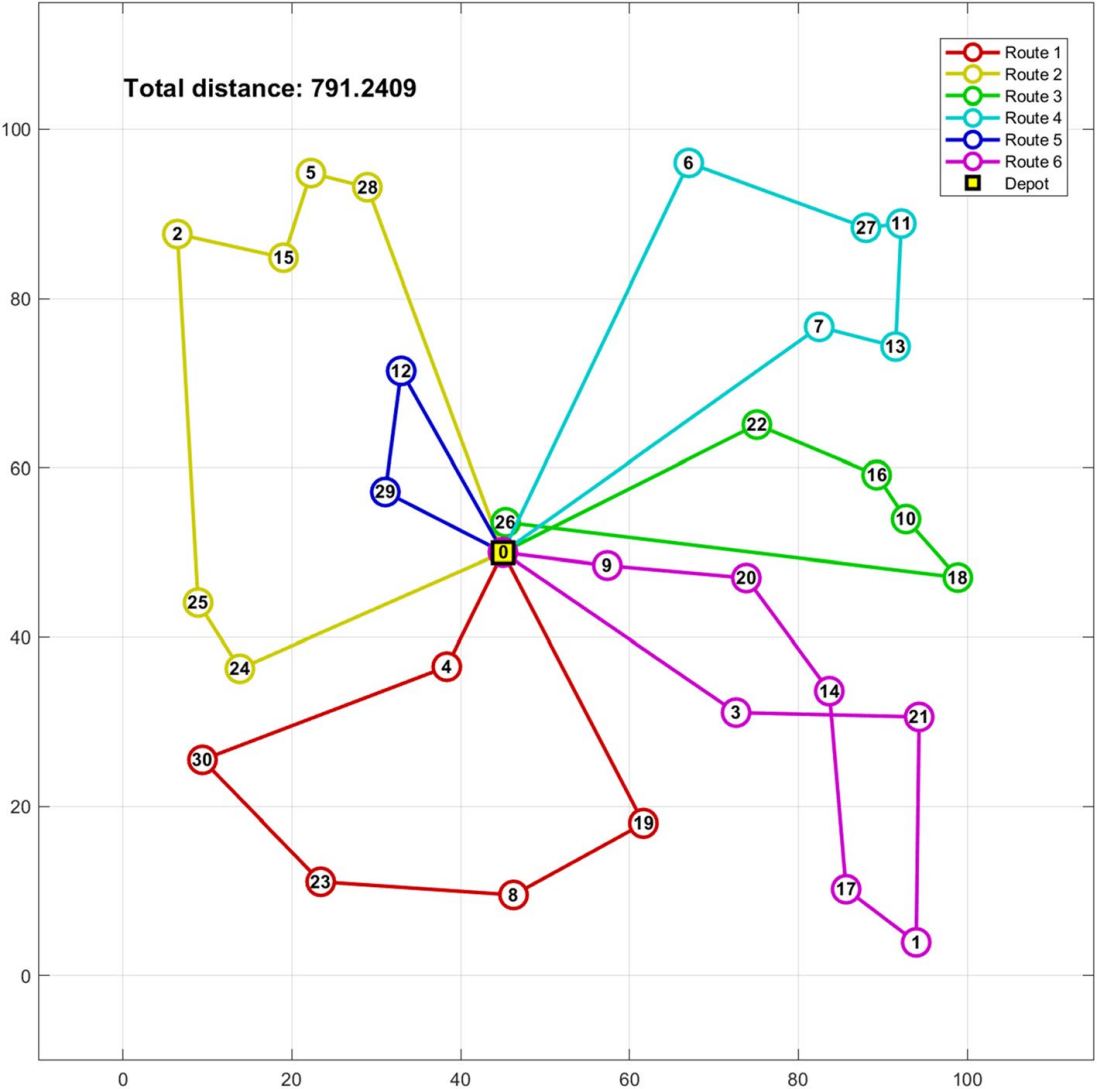
Best solution for the CVRP of 30-customer problem.

Table 21 consolidates the performance metrics of the different algorithms tested. Significantly, the hGWOA algorithm emerged as the frontrunner, with its best %dev solution outperforming other optimization techniques: WOA by 20.2%, DA by 31.8%, ALO by 36.6%, and GWO by 19.5%. Figure 13 provides a visual comparison, highlighting the superior data distribution of hGWOA compared to other algorithms. This analysis reaffirmed the effectiveness of the hGWOA algorithm in optimizing delivery routes, achieving a cumulative distance of 791.24 units. The delivery routes for the six trucks, as determined by hGWOA, are delineated in Table 22 and further illustrated in Fig. 14.

**Table 21:** Results of different algorithms on 30-customer problem.

Algorithm	Scattering of optimal solutions					Max	Min	Mean	Deviation ratio of the best solution
hGWOA	816.86	792.34	889.81	816.86	791.24	974.48	791.24	852.92	0.0%
	896.85	841.10	909.75	911.02	864.35				
	832.85	898.49	974.48	814.37	795.57				
	824.74	901.12	791.24	895.56	799.85				
WOA	973.10	950.88	1023.60	1031.80	1027.80	1092.80	950.88	1024.84	20.2%
	1082.20	1086.10	1008.10	959.50	1033.37				
	1007.78	1064.80	1061.40	1038.20	1092.80				
	996.51	962.57	993.40	1049.90	1053.00				
DA	1146.70	1151.60	1160.70	1190.70	1155.50	1300.90	1042.49	1192.33	31.8%
	1202.50	1255.97	1263.80	1193.67	1079.10				
	1226.60	1288.90	1264.90	1160.60	1130.00				
	1250.10	1042.49	1177.80	1300.90	1204.10				
ALO	1166.60	1280.20	1080.60	1225.00	1167.80	1303.20	1080.60	1184.07	36.6%
	1170.41	1138.40	1214.80	1215.40	1131.40				
	1227.20	1303.20	1098.90	1112.30	1243.70				
	1197.00	1178.80	1151.80	1158.90	1218.90				
GWO	1157.40	1046.10	1119.50	1103.60	945.47	1159.95	945.47	1061.99	19.5%
	816.86	1128.40	1030.20	1054.40	1064.70				
	896.85	1141.70	1107.70	1012.10	993.68				
	832.85	1104.00	1041.20	1035.70	1159.95

**Table 22:** Routing of vehicles and distance using hGWOA algorithm on 30-customer problem.

Routes of the vehicles on 30-customer problem		Distance
Route 1	0- > Customer 19- > Customer 8- > Customer 23- > Customer 30- > Customer 4- > 0	142.6
Route 2	0- > Customer 24- > Customer 25- > Customer 2- > Customer 15- > Customer 5- > Customer 28- > 0	163.2
Route 3	0- > Customer 22- > Customer 16- > Customer 10- > Customer 18- > Customer 26- > 0	122.05
Route 4	0- > Customer 7- > Customer 13- > Customer 11- > Customer 27- > Customer 6- > 0	147.44
Route 5	0- > Customer 12- > Customer 29- > 0	54.64
Route 6	0- > Customer 3- > Customer 21- > Customer 1- > Customer 17- > Customer 14- > Customer 20- > Customer 9- > 0	161.31
Total distance: 791.24 units

For the computational studies, algorithms were developed in Java and executed on a personal computer powered by an Intel(R) Core(TM) i7-1165G7 processor operating at 2.80 GHz. Each algorithm was subjected to 20 runs, using 60 search agents, and covered 200 iterations for every CVRP test scenario.

## Conclusion

This study unveils a novel approach to global optimization by merging the WOA method with GWO techniques. This strategic combination aims to seamlessly merge the exploratory capabilities of WOA with the search space exploitation proficiencies inherent to GWO, targeting optimal outcomes. The resulting hybrid algorithm, termed hGWOA, has been meticulously assessed using both classical test functions and CEC2017 benchmark test functions. The empirical results underscore hGWOA’s marked advantage over both GWO and WOA in achieving global optimization.

Additionally, this research employs the innovative hGWOA algorithm to tackle the Routing Logistics Challenge faced by limited-capacity cement trucks, referred to as the CVRP. Through computational evaluations across various contexts—namely, two unique case studies and a practical project—it is evident that hGWOA excels in crafting high-quality solutions to CVRP optimization issues. Based on these findings, hGWOA emerges as a promising meta-heuristic approach, suitable not only for the CVRP dilemma but also for a spectrum of related optimization challenges.

## Directions for future research

This study emphasizes the application of the hGWOA method specifically to address CVRP issues. However, in real-world materials transportation scenarios, VRP challenges often encompass a myriad of factors, including delivery timelines, carbon emissions, fuel consumption metrics, and prevailing road traffic conditions. It is therefore anticipated that subsequent research endeavors will deploy the hGWOA methodology to grapple with intricate and multifaceted VRP conundrums that simultaneously align with customer stipulations.

Upon comparative evaluation with established swarm-based optimization algorithms, specifically DA, ALO, WOA, and GWO, the hGWOA paradigm manifests a commendable balance between exploration and exploitation capacities. Moreover, it showcases competitive prowess across diverse magnitudes of the CVRP. A limitation, however, arises when scaling to larger problem sets, wherein hGWOA occasionally grapples with local optimization pitfalls. As a forward-looking initiative, forthcoming research aims to concoct a composite model wherein hGWOA operates synergistically with ancillary techniques. These might encompass adaptive weighting customizations, Yin-Yang-centric learning mechanisms, mutation procedures, and crosstalk interventions. Such an integrative approach aims to bolster hGWOA's effectiveness in navigating optimization challenges, particularly within transportation management, and extending to broader technical spheres.

The hybrid model hGWOA may converge slowly, especially when dealing with high-dimensional or complex optimization problems. Employ techniques such as adaptive parameter settings, dynamic population sizing, or hybridization with other optimization algorithms to accelerate convergence and improve efficiency. In addition, the performance of this model may deteriorate when applied to extremely large-scale optimization problems. Hence, future research could consider implementing problem-specific adaptations, parallel processing, or divide-and-conquer strategies to make hGWOA more suitable for handling larger problem instances.

## Data Availability

Some or all data, models, or code that support the findings of this study are available from the corresponding author upon reasonable request.
